# Proline Isomerization and Molten Globular Property of TgPDCD5 Secreted from *Toxoplasma gondii* Confers Its Regulation of Heparin Sulfate Binding

**DOI:** 10.1021/jacsau.3c00577

**Published:** 2024-03-20

**Authors:** Gloria Meng-Hsuan Lin, Tsun-Ai Yu, Chi-Fon Chang, Chun-Hua Hsu

**Affiliations:** † Department of Agricultural Chemistry, National Taiwan University, Taipei 10617, Taiwan; ‡ Genome and Systems Biology Degree Program, National Taiwan University and Academia Sinica, Taipei 10617, Taiwan; § Genomic Research Center, Academia Sinica, Taipei 115201, Taiwan; ∥ Institute of Biochemical Sciences, National Taiwan University, Taipei 115201, Taiwan

**Keywords:** *Toxoplasma gondii*, molten globule, Heparan/heparin sulfate binding, Proline isomerization, NMR structure

## Abstract

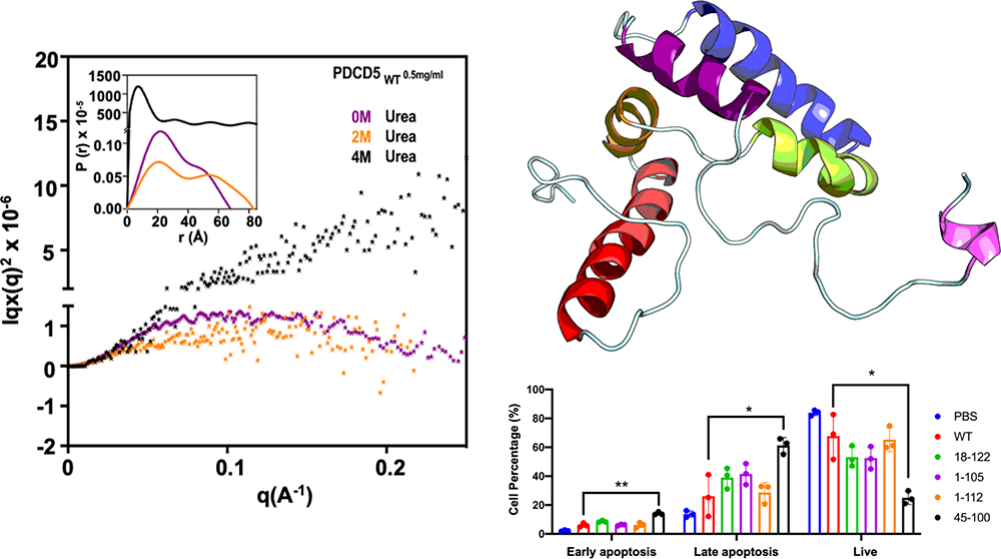

Toxoplasmosis, caused by *Toxoplasma gondii*, poses risks to vulnerable populations. TgPDCD5, a secreted protein of *T. gondii*, induces apoptosis through heparan sulfate-mediated endocytosis. The entry mechanism of TgPDCD5 has remained elusive. Here, we present the solution structure of TgPDCD5 as a helical bundle with an extended N-terminal helix, exhibiting molten globule characteristics. NMR perturbation studies reveal heparin/heparan sulfate binding involving the heparan sulfate/heparin proteoglycans-binding motif and the core region, influenced by proline isomerization of P107 residue. The heterogeneous proline recruits a cyclophilin TgCyp18, accelerating interconversion between conformers and regulating heparan/heparin binding. These atomic-level insights elucidate the binary switch’s functionality, expose novel heparan sulfate-binding surfaces, and illuminate the unconventional cellular entry of pathogenic TgPDCD5.

## Introduction

*Toxoplasma gondii*, a protozoan parasite of the *phylum* Apicomplexa, poses significant threats to public health and the economy by infecting various warm-blooded animals, including humans. *T. gondii* infection can lead to severe diseases such as encephalitis and retinochoroiditis, particularly in immunocompromised patients. Additionally, infection during pregnancy can result in abnormal pregnancy outcomes and fetal hydrocephalus.^1−3^ Current treatment options for this widespread infection are limited and ineffective for pregnant women, newborns, and infants.^4−6^ Therefore, the urgent need for novel antiparasitic agents against *T. gondii* arises due to the challenges associated with eliminating this epidemic.

During *T. gondii* infection, the parasite undergoes stage conversion between rapidly proliferating tachyzoite and slowly replicating bradyzoite. The tachyzoite triggers acute toxoplasmosis, while the encysted bradyzoite leads to chronic latent infection. *T. gondii* is an obligate intracellular pathogen, and the host deploys innate immunity as a defense mechanism against intracellular infection. However, the pathogen has evolved strategies to evade host innate immunity by secreting proteins into the host cell cytosol, nucleus,^7−9^ and even contacting but not invading bystander cells.^10−13^ Notably, one consequence of the modulation by *T. gondii*-secreted proteins is the regulation of host cellular fate through apoptosis pathways.^14,15^ Accumulating evidence shows that *T. gondii* induces host cell apoptosis to facilitate tissue penetration and dampen host immunity, particularly in fibroblasts and innate immune cells.^8,16,17^ Among the secreted proteins, TgPDCD5 (*T. gondii* programmed cell death protein 5) has been reported to possess the ability to induce apoptosis in human promyeloblastic cells and mouse macrophages.^16,18^ Interestingly, TgPDCD5 secreted by GT1 strain (Gene TGGT1_207690, studied in this project) lacks a signal peptide sequence, yet previous studies have detected its presence in the culture medium of GT1 parasite-infected cells, suggesting a secretory mechanism that remains elusive.^16^

Based on the presence of a heparan sulfate/heparin proteoglycans (HSPG) binding motif (KVTMRR, residues 108–113) at the C-terminus of TgPDCD5, it was proposed that the induction of host cell apoptosis occurs through endocytosis mediated by the interaction between TgPDCD5 and HSPG.^18^ In this study, we elucidate the biophysical features and further determine the solution structure of TgPDCD5 as a dynamic molten globule (MG). Additionally, we uncover the binding of TgPDCD5 to heparin sulfate and its regulation through a proline switch. These findings pave the way for a better understanding of the structure and mechanism of TgPDCD5′s preapoptotic effects on host cells.

## Results and Discussion

### TgPDCD5 Possesses Helical Characteristics with Molten Globular Property

**Figure 1:**
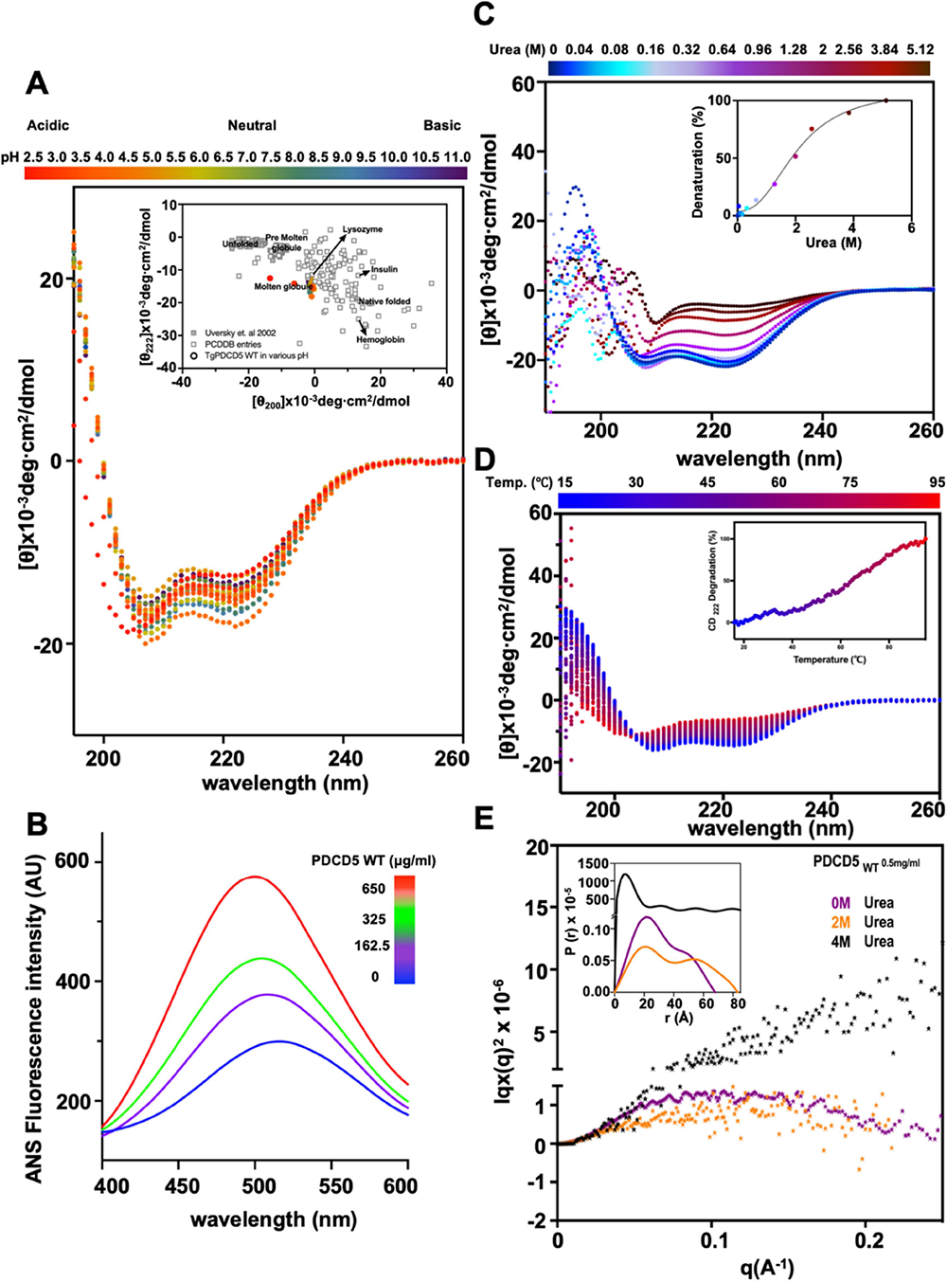
Molten globular behavior of TgPDCD5. (A) CD spectra of TgPDCD5 under different pH conditions. The inset shows a double wavelength plot obtained from CD spectra at different pH values compared with the reference spectra recorded in the web server CAPITO. (B) ANS-fluorescence assay profile of TgPDCD5. Blue signals represent ANS alone, while purple, green, and red signals represent ANS incubated with different amounts of protein. (C) CD spectra of TgPDCD5 incubated with varying concentrations of urea. The inset shows the chemical unfolding of the protein, monitored by CD spectrometry at 222 nm. (D) Thermal denaturation of TgPDCD5 was monitored by CD spectrometry. The inset shows the thermal unfolding of the protein monitored at 222 nm. (E) Kartky-Porod plot of TgPDCD5 chemical unfolding by urea based on SAXS data. The *P*(*r*) vs *r* profiles from the data are inserted.

To determine the structural characteristics of TgPDCD5, circular dichroism (CD) measurements were conducted under various chemical conditions. The CD spectra of TgPDCD5 at different pH values exhibited two negative bands at 222 and 208 nm and a positive band at 195 nm, indicative of an α-helical structure (Figure 1A). Surprisingly, the spectra of TgPDCD5 remained nearly identical even under acidic or basic conditions. Utilizing the CAPITO (CD Analysis and Plotting Tool) server^19^ to predict the fold states, all pH conditions suggested that TgPDCD5 adopts a molten globular intermediate state (Figure 1A).

To investigate the molten globular properties further, the exposure of hydrophobic regions in native TgPDCD5 was assessed using the fluorophore dye 8-anilino-1-naphthalenesulfonic acid (ANS). The addition of TgPDCD5 resulted in a blueshift and increased fluorescence signal of ANS, indicating the presence of solvent-exposed nonpolar hydrophobic sites, a characteristic of a molten globular protein (Figure 1B). Furthermore, the unfolding process of TgPDCD5, monitored by CD spectra in the presence of urea as a denaturing agent, revealed a gradual loss of the secondary structure, suggesting the absence of a compact structure (Figure 1C). Similar unfolding patterns were observed in the CD spectra of TgPDCD5 at increasing temperatures (Figure 1D). Notably, the absence of a sigmoidal transition curve for defining a melting temperature (*T*_m_) indicated that TgPDCD5 lacks an unfolding threshold, which is typical of a globular protein (inset in Figure 1D). These findings demonstrate that TgPDCD5 predominantly adopts a helical structure without a rigid three-dimensional conformation.

To further investigate the molten globular behavior of TgPDCD5, we employed small-angle X-ray scattering (SAXS) in solution. SAXS is a technique that provides information about the size, compactness, and shape of macromolecules in scattering experiments. We conducted SAXS experiments under the following conditions: native state (absence of urea), midpoint of unfolding (2 M urea), and fully unfolded protein (4 M urea). The SAXS data were represented by the Kratky–Porod plot (Figure 1E). In the native state, the Kratky curve displayed a smeared hill-shaped pattern, with a flat top around *q* 0.08–0.15 Å^–1^, indicating the flexibility of the protein and the linkage between two subunits. The estimated radii of gyration (Rg) values for the subunits were 23.9 and 50.8 Å (inset in Figure 1E). When TgPDCD5 was incubated with 2 M urea, the Kratky curve became flatter, exhibiting a horizontal divergence near zero. Furthermore, SAXS data for TgPDCD5 incubated with 4 M urea displayed a hyperbolic shape, characteristic of a fully unfolded particle. The observed changes in the SAXS data were consistent with gradual chemical denaturation observed in the CD profiles (Figure 1C). These pieces of evidence support the conclusion that TgPDCD5 adopts a molten globular protein state.

### NMR Solution Structures of TgPDCD5 in Both Cis- and Trans-Forms

The backbone and side-chain assignments of TgPDCD5 (Figure S1) were completed to 96% under the experimental conditions (25 mM phosphate buffer pH 4.5 with 100 mM NaCl at 310 K) and have been deposited in the Biological Magnetic Resonance Data Bank (http://www.bmrb.wisc.edu ) with the accession number 28099.^20^ Due to the presence of proline residues in the TgPDCD5 sequence, paired backbone amide signals of cis-/trans-form of residues (E4, E5, T106, K108, V109, T110, and M111) could be assigned on the HSQC spectrum. The ^1^H-^15^N HSQC spectrum of TgPDCD5 exhibited dispersed resonances with slight cloudiness in the range of 7.7–8.4 ppm (Figure S1), indicating the presence of a partially unstructured region and supporting the molten globular state of the protein.

The HSPG-binding motif of TgPDCD5 was identified at the C-terminus (residues 108–113), which was predicted to be an unstructured loop.^20^ Notably, the presence of a proline residue (P107) adjacent to this binding motif led us to speculate that HSPG binding in TgPDCD5 may be regulated by proline cis–trans isomerization, acting as a switch. To delve into this aspect, the nuclear magnetic resonance (NMR) solution structure of TgPDCD5 was determined using CYANA3. In addition to the relevant dihedral angle (omega angle) for cis and trans prolines, relative assignments of cis-/trans-form of residues (E4, E5, T106, K108, V109, T110, and M111), coupled with relative dihedral angles, were employed to artificially distinguish between the trans- and cis-forms of TgPDCD5. Comprehensive structural statistics of the NMR ensembles of TgPDCD5 is given in Table 1.

**Table 1:** Restraints and Structure Statistics for 20 Lowest Energy Conformers of TgPDCD5 Trans- and Cis-Major States

parameter	value	
	trans-major	cis-major
number of distance restraints		
total NOEs	829	728
short range (|*i*–*j*| = 1)	625	576
medium range (1 < |*i*–*j*| < 5)	111	78
long range (|*i*–*j*| > 5)	93	74
number of dihedral angle restraints		
φ angle	41	46
ψ angle	46	46
number of restraint violations (maximum violation)		
distance restraint violations >0.2 Å	0	0
dihedral angle restraints >5°	0	0
PROCHECK-NMR Ramachandran map analysis (%)		
most favored regions	85.0	75.2
additional allowed regions	13.3	20.4
disallowed regions	1.77	4.4
RMSD (Å)		
helix α1 (residues 18–33)	0.95 ± 1.17^a^	1.13 ± 0.65^a^
	1.92 ± 1.24^b^	1.98 ± 0.71^b^
helix α2 (residues 36–45)	0.60 ± 0.27^a^	0.44 ± 0.32^a^
	1.89 ± 0.40^b^	1.37 ± 0.36^b^
helical bundle region (residues 49–97)	1.89 ± 0.78^a^	1.43 ± 0.49^a^
	2.53 ± 0.74^b^	2.00 ± 0.44^b^
secondary structure region of the helical bundle α3−α5 (residues 49–58; 62–78; 87–97)	1.66 ± 0.76^a^	1.34 ± 0.50^a^
	2.24 ± 0.69^b^	1.90 ± 0.45^b^

a For backbone.

b For all heavy atoms.

**Figure 2:**
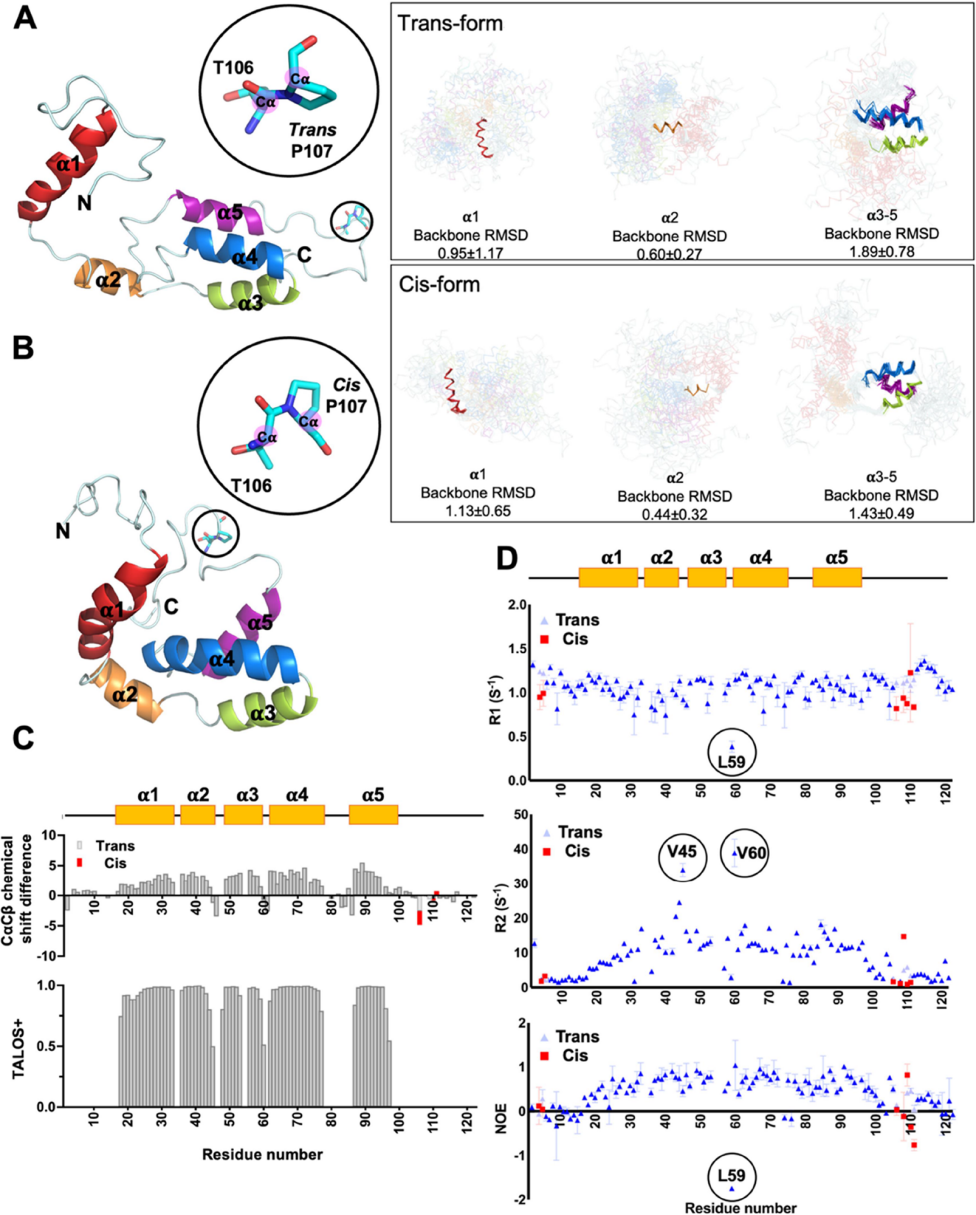
Solution structure and dynamics of TgPDCD5. The structures of TgPDCD5 with trans-form P107 (A) or with cis-form P107 (B) are depicted. The relative selected conformer with trans-P107 (A) or cis-P107 (B) is presented in the left panel as a representation, while the ensemble of the 20 lowest energy NMR-derived structures of TgPDCD5 with trans-P107 (A) or cis-P107 (B) is displayed in the right panel. The secondary structures of TgPDCD5, including helix α1, helix α2, helix α3, helix α4, and helix α5, are colored red, orange, green, blue, and deep purple, respectively. Due to its molten globular feature, solution conformers of TgPDCD5 can only be superimposed by the secondary structure elements: helix α1, helix α2, and helices α3−α5, rather than the full length. The relative RMSD numbers are labeled. (C) Secondary structures of TgPDCD5 are based on the Cα and Cβ chemical-shift difference and the TALOS+ prediction. (D) NMR dynamics information for TgPDCD5. The ^15^N-relaxation parameters (*R*_1_, *R*_2_, NOE) are presented. Residues in the trans-form are indicated by blue triangles, and residues in the cis-form are indicated by red squares.

Two ensembles, each comprising the 20 lowest energy NMR-derived solution structures of TgPDCD5 with trans-P107 and cis-P107 (Figure 2A,B, respectively), were generated and deposited with PDB accession codes: 8I25 and 8I26. The structures revealed a heterogeneous ensemble of flexible conformations that could not be superimposed by full-length proteins, highlighting its molten globular feature. Through secondary structure-defined superimposition, the five helices of TgPDCD5 could be classified into a core-helical bundle region and two dissociated helices. Detailed structural statistics of the NMR ensembles of TgPDCD5 are summarized in Table S1.

In the solution structure ensembles as depicted (Figure 2A,B), the overall structure of TgPDCD5 comprised five α-helices (α1: 18–33; α2: 36–45; α3: 49–58; α4: 62–78; and α5: 87–97) connected by long loops. This structural arrangement aligns with the TALOS+ secondary structural prediction, as indicated by the Cα-Cβ chemical-shift difference (Figure 2C). The α1 helix was found to be a distinct structural region, as evidenced by the absence of observed nuclear Overhauser effect (NOE) interactions with the rest of the TgPDCD5 structure. On the other hand, helices α3-α5 formed a triple-helical bundle, with helix α2 connected to this bundle via the short linker Lα2α3 (L46-A49). The N- and C-terminal segments (1–17 and 98–122, respectively) were observed to be disordered in structure.

The residual dipolar coupling (RDC) numbers measured with filamentous phages as the alignment media were used to investigate the orientation of amide vectors of molten globular TgPDCD5 (Figure S2 as measured RDCs). The overall amide RDCs throughout the helices α1, α2, α3, and α5 were negative but positive for α4 helix suggesting the orientation of α4 helix amide vectors is in the opposite direction, as shown in Figure2.

### NMR Dynamics of the Molten Globular TgPDCD5

MGs are recognized for their dynamic nature.^21−23^ To unveil flexibility at the pico-nanosecond time scale, we conducted NMR backbone relaxation measurements (Figure 2D), and the dynamics parameters derived from reduced spectral density mapping were obtained (Figure S2). According to our heteronuclear NOE data, the N- and C-terminal segments exhibited low values on the NOE, indicating higher flexibility compared to the rest of TgPDCD5, which displayed ordered secondary structures. Notably, residue L59 exhibited a negative NOE value, indicating rapid motion at the subnanosecond time scale. On the other hand, the residue with the highest *R*_2_ is V60, and increasing R_2_ values were observed at the end of α2 (R43-V45). According to the reduced spectral density function, significant increases in *J*(0) values at V45 and V60 suggest the potential chemical exchange processes.

Within the α3−α5 helical bundle, which contains 24 hydrophobic amino acids, a loosely packed hydrophobic core of TgPDCD5 is presumed to form. However, residue L59, located between α3 and α4, exhibits highly dynamic properties. Additionally, its neighboring residue V60 might undergo a chemical exchange process. Based on these intramolecular dynamics information, we suspect that even the “core region” helical bundle α3−α5 is nonrigid but loosely packed.

To gain further insight, the thermal and urea-induced denaturation of a fragment spanning amino acids 45–100 (TgPDCD5_45–100_), which covers the “core region” α3−α5, was monitored using CD signals at 222 nm (Figure S3A,B). The results demonstrated the gradual unfolding of TgPDCD5_45–100_ with increasing temperature and urea concentration. The Kratky curve of native TgPDCD5_45–100_ displayed a smeared shape with a flat plateau at q values above 0.08 Å^–1^, reflecting its high flexibility and a significant radius of gyration (Rg) value of approximately 20 Å (Figure S3C,D). Interestingly, SAXS data for TgPDCD5_45–100_ incubated with 2 and 4 M urea exhibited hyperbolic curves, characteristic of fully unfolded particles (Figure S2C). However, CD signals at 222 nm indicated the partial denaturation of TgPDCD5_45–100_ in the presence of 2 M urea (Figure S3B). The presence of a loosely packed hydrophobic core, a reported characteristic feature of MGs,^21,23,24^ is likely attributed to the α3−α5 helical bundle region.

### Core Region α3−α5 of TgPDCD5 Participate the Interaction with Heparin Sulfate

To investigate the crucial fragment of TgPDCD5 responsible for its apoptosis-inducing ability mediated by HSPG binding, we designed and constructed several truncations of TgPDCD5, including protein fragments 18–122, 1–105, 1–112, and 45–100. The appropriate protein concentrations of purified full-length TgPDCD5 and the truncated forms were determined by using the MTT assay on human monocyte U937 cells (Figure S4A). Subsequently, the levels of apoptosis in cultured cells treated with purified proteins at a concentration of 10 μg/mL for 24 h were assessed using Annexin-V/PI staining assay and flow cytometry (Figure S4B).

Surprisingly, the apoptosis-inducing levels of the TgPDCD5 fragments TgPDCD5_18–122_ and TgPDCD5_1–112_, which retained the C-terminal segment, were comparable to those of full-length TgPDCD5. Interestingly, even in the absence of the C-terminal HSPG-binding motif, TgPDCD5_1–105_ exhibited a slightly lower level of apoptosis induction but with no significant difference compared to full-length TgPDCD5. Additionally, TgPDCD5_45–100_, which contained only the core region, retained the ability to efficiently induce apoptosis in U937 cells. These findings suggest that the core region of TgPDCD5 alone is sufficient for interaction with HSPG. Thus, it implies that TgPDCD5 may employ mechanisms beyond the C-terminal HSPG-binding motif to interact with heparan/heparin sulfate proteoglycans.

Heparan sulfate is a ubiquitous extracellular matrix component found in multicellular animals, whereas heparin is produced by mast cells and is involved in pathogen recognition.^25−27^ Both heparan sulfate and heparin are sulfated polysaccharides composed of repeating disaccharide units. Heparin, with a smaller molecular weight average of 20 kDa, is more highly sulfated and charged compared to heparan sulfate, making it the immune system-specific and more sulfated variant of heparan sulfate.^27,28^ Given these properties, heparin was selected for further biophysical analysis.

**Figure 3:**
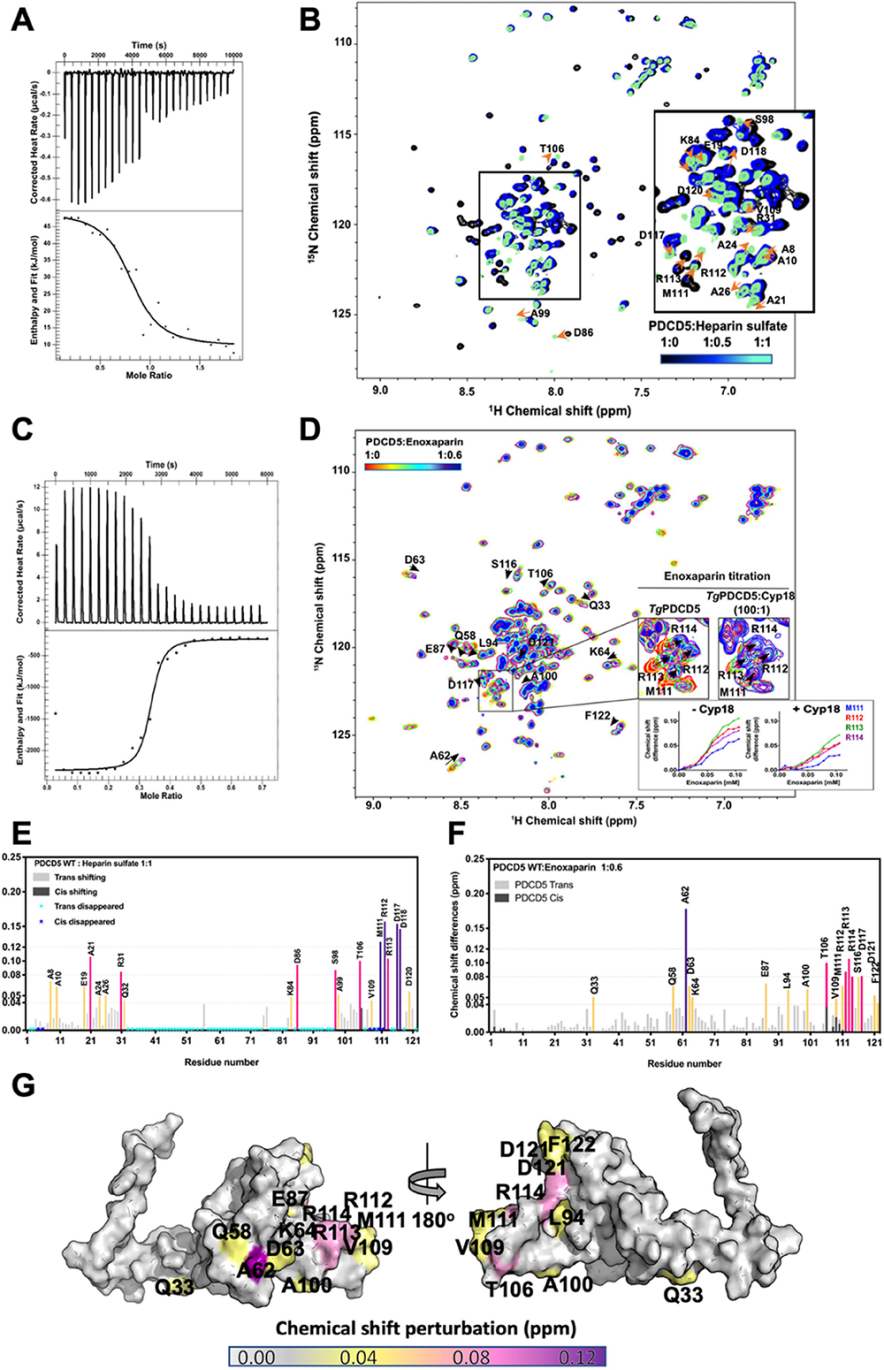
Interactions with heparin sulfate polysaccharide determined by ITC and NMR. (A) Isothermal titration calorimetry analysis of TgPDCD5 titrated with heparin sulfate. Upper panel: raw data in μcal/s versus time, showing heat release during titration. Lower panel: integration of raw data yielding the heat per mole versus molar ratio. (B) Overlay of 2D ^1^H-^15^N HSQC spectrum of TgPDCD5 titrated with heparin sulfate. (C) Isothermal titration calorimetry analysis of TgPDCD5 titrated with Enoxaparin. Upper panel: raw data in μcal/s versus time showing heat release during titration. Lower panel: integration of raw data yielding the heat per mole versus molar ratio. (D) Overlay of the 2D ^1^H-^15^N HSQC spectrum of TgPDCD5 titrated with Enoxaparin. (E) Chemical-shift differences of each residue measured from heparin sulfate titration are shown. Residues with significant changes are labeled. Amino acids with broadened backbone amide signals are marked with stars. (F) Chemical-shift differences of each residue measured from Enoxaparin titration are shown. Residues with significant changes are labeled. Amino acids with broadened backbone amide signals are marked with stars. (G) Mapping of residues with significant chemical-shift changes onto the NMR solution structure of trans-form TgPDCD5 during Enoxaparin titration. Residues with CSPs between 0.04 and 0.08 ppm are colored in yellow, CSPs between 0.08 and 0.12 ppm are colored in magenta, and CSPs over 0.12 are colored in purple.

To investigate the binding ability of TgPDCD5 to heparin, we performed isothermal titration calorimetry (ITC) experiments. The results revealed a strong interaction between TgPDCD5 and heparin with a dissociation constant (*K*_d_) of approximately 1.84 μM (Figure 3A, Table 2). To gain more insights into the interaction, we conducted a series of NMR experiments. The NMR chemical-shift perturbation assay demonstrated significant line-width changes in the HSQC peaks of residual backbone amides upon the addition of heparin sulfate, particularly those intensities dropping then disappeared peaks that corresponded to amino acids (Figure 3B) located in the core region of the protein (Figure 3E, cyan). These vanishing signals indicate an interaction occurring in the intermediate exchange regime and may also be attributed to the slower tumbling behavior of the larger TgPDCD5-heparin sulfate complex.

**Table 2:** Thermodynamics of Heparin Sulfate or Enoxaparin Binding of TgPDCD5^a^

TgPDCD5 titrates	*K*_d_ (μM)	*n*	deltaG	deltaH (kJ/mol)	deltaS (J/mol.K)	–*T*Δ*S*	exo/endothermic
heparin sulfate	0.91 ± 1.12	0.77 ± 0.03	–35.80 ± 1.90	46.71 ± 5.94	266.17 ± 17.33	–82.51 ± 5.37	exothermic
enoxaparin	0.07 ± 0.10	0.31 ± 0.02	–43.15 ± 2.82	–1779.67 ± 261.58	–5601.67 ± 834.76	–1736.52 ± 258.77	endothermic

a The analytic numbers are the average of three repeats.

To overcome this challenge, we utilized a shorter polymer fragment of heparin sulfate, called Enoxaparin, for our investigation. Our ITC experiments revealed a strong interaction between TgPDCD5 and Enoxaparin, with a dissociation constant (*K*_d_) of approximately 0.105 μM (Figure 3C, Table 2). Since heparin sulfate and Enoxaparin consist of repeating disaccharide units, we applied a one-site-binding model to our ITC data, albeit with a noncanonical molar ratio, indicating that multiple TgPDCD5 molecules interact with a single Enoxaparin polymer. Interestingly, the binding of Enoxaparin to TgPDCD5 was found to be endothermic and driven by a strong enthalpy (Figure 3C), similar to previous reports of protein–heparin interactions.^29−32^

In contrast, the titration of heparin displayed exothermic behavior (Figure 3A). This difference in thermodynamic profiles may be attributed to the propensity of high-molecular-weight heparin polymers to form nanoparticles in solution, which can undergo disassembly or conformational changes, resulting in observed exothermicity. Furthermore, NMR perturbation analysis upon Enoxaparin titration revealed significant shifts in several HSQC peaks (Figure 3D,F), located attractively in the core region beside the C-terminal of TgPDCD5 (Figure 3G). Notably, the resonance of the A62 residue exhibited the most pronounced chemical-shift difference upon the addition of Enoxaparin (Figure 3F,G).

### Proline Isomerization Regulating the Heparin Interaction of TgPDCD5

In the backbone amide assignment of TgPDCD5 (Figure S1), a set of weak peaks, including those corresponding to cis-form proline residues (E4′ and E5′ following proline residue P3, as well as T106′, K108′, V109′, T110′, and M111′ related to P107), were observed. Notably, the minor peaks related to the P107 residue include the HSPG-binding motif (108–113) of TgPDCD5. Typically, the trans-form of proline is more thermodynamically stable than the cis-form, requiring crossing a free energy barrier of approximately 15.6 kcal/mol for the transformation from trans to cis proline.^33^ This transformation of cis/trans isoforms necessitates the involvement of enzymes called peptidyl-prolyl isomerases to accelerate the process. These observations led us to speculate that proline isomerization may function as a switch to regulate the binding of heparin sulfate molecules, potentially modulated by peptidyl-prolyl isomerases from either the parasite or the host. Interestingly, TgPDCD5 shares a secretion time frame with a cyclophilin homologue named TgCyp18, secreted by the tachyzoite stage of *T. gondii*.^34,35^ Moreover, cyclophilins belong to a group of enzymes known to accelerate the peptidyl-prolyl cis/trans isomerization process.^36^

**Figure 4:**
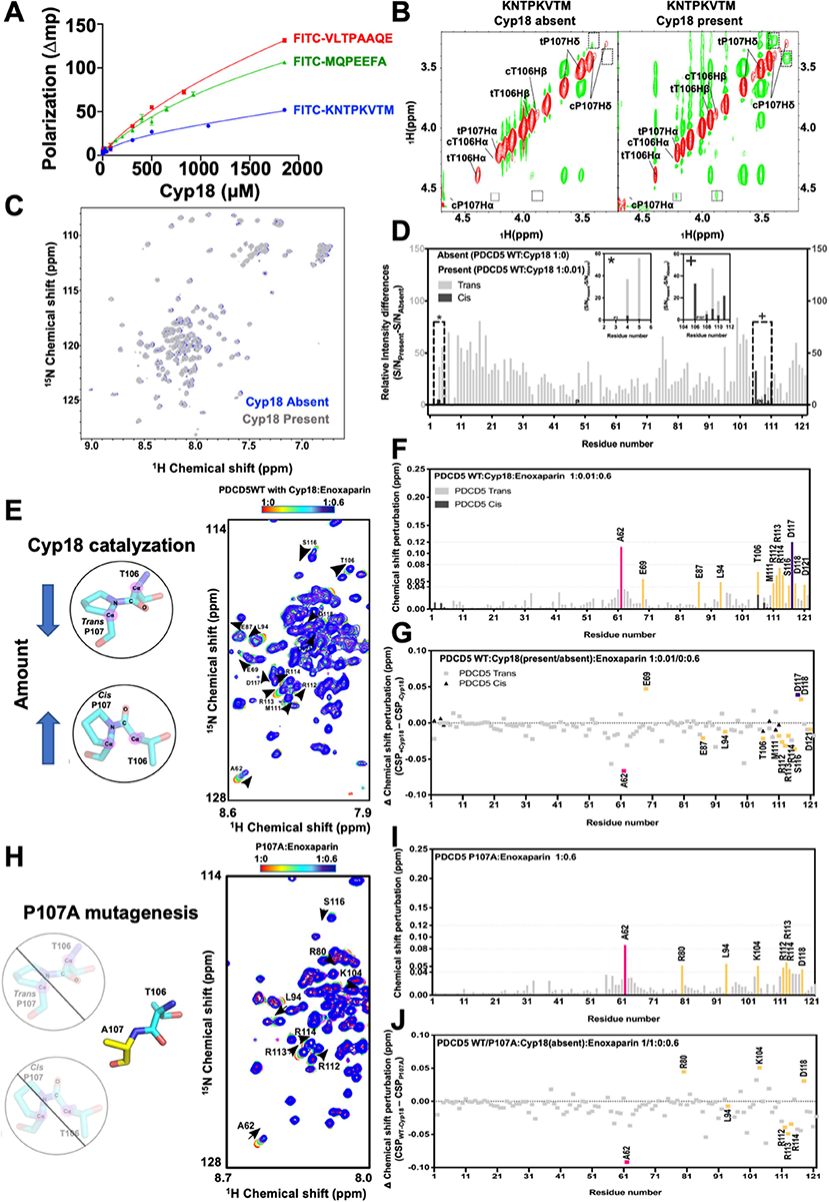
Isomerization of P107 regulates the interaction between TgPDCD5 and polysaccharides. (A) Binding affinities of the interaction between TgCyp18 and 150 μM C-terminal peptide (FITC-KNTPKVTM), middle-position peptide (FITC-VLTPAAQE), and N-terminal peptide (FITC-MQPEEFA) are derived from a one-site binding model using Prism. (B) ^1^H-^1^H ROESY spectrum at 298 K of the C-terminal peptide with the absence (ratio 40:0) and presence (ratio 40:1) of enzyme Cyp18 is shown in the left and right panel, respectively. The positive peaks are colored in red and negative peaks in green. The ^1^H resonances of T106 and P107 are marked. The resonances from the cis and trans forms are labeled as c and t, respectively. The dashed-line boxes indicate the signals indicating accelerations of the P107 peptidyl isomerization from the presence of the ROE signals of neighboring hydrogens. (C) Overlay of ^1^H-^15^N HSQC spectra of TgPDCD5 treated with TgCyp18. (D) Signal intensity difference of TgPDCD5 treated with TgCyp18. Paired backbone amide-signal intensity differences of cis-/trans-form residues (E4, E5, T106, K108, V109, T110, and M111) are highlighted by inset plots (*, + in D). (E) Illustration of TgCyp18 catalyzing the isomerization of TgPDCD5, and the overlay of ^1^H-^15^N HSQC spectra of Enoxaparin titrating TgPDCD5 with TgCyp18 at selected region. (F) Chemical-shift difference of TgPDCD5 while Enoxaparin is titrated in the presence of TgCyp18. (G) Delta chemical-shift difference of TgPDCD5, subtracting CSPs from titrations with the absence of TgCyp18 from the CSPs from titrations with the presence of TgCyp18. (H) Illustration of P107A mutation in TgPDCD5, and the overlay of ^1^H-^15^N HSQC spectra of Enoxaparin titrating TgPDCD5 P107A at selected region. (I) Chemical-shift difference of TgPDCD5 P107A while Enoxaparin is titrated. (J) Delta chemical-shift difference of TgPDCD5, subtracting CSPs from titrations with the absence of TgCyp18 toward TgPDCD5 WT to the CSPs from titrations toward P107A. Residues with CSPs over 0.12 are colored in purple. Residues with CSPs in the range of 0.08–0.12 and 0.04–0.08 ppm are colored strawberry pink and yellow, respectively. The delta chemical-shift difference represents the CSPs determined in the presence of TgCyp18 or P107A subtracted from the CSPs determined in the absence of TgCyp18.

To investigate the molecular function and substrate specificity of TgPDCD5, we examined the binding affinities and activity of purified recombinant TgCyp18 toward three peptidyl-prolines (P3, P48, and P107) within TgPDCD5. Fluorescein isothiocyanate (FITC)-labeled peptides corresponding to the N-terminus (N-peptide, ^1^MQPEEFA^7^), middle-position (M-peptide, ^45^VLTPAAQE^52^), and C-terminus (C-peptide, ^104^KNTPKVTM^111^) of TgPDCD5 were chemically synthesized. Fluorescence polarization (FP) experiments were performed, revealing dissociation constants of 1405, 1198, and 311.3 μM for the N-, M-, and C-peptides, respectively (Figure 4A). As expected, TgCyp18 exhibited the highest binding affinity toward the C-peptide among the three proline-containing peptides.

To provide evidence of the catalytic activity of TgCyp18 on the C-peptide, we utilized NMR rotating-frame Overhauser enhancement spectroscopy (ROESY). The diagonal peaks (shown in red) corresponding to the cis and trans conformations resonance assignments of TgPDCD5 C-peptide (^104^KNTPKVTM^111^), which had been completed before the ROESY assay. Analysis of the ROESY spectra in the presence of TgCyp18 revealed the appearances of the ROE signals (shown in green) between neighboring hydrogens of the cis-form P107, including pairs between cis-form T106 H_α_ and P107 H_α_, cis-form T106 H_β_ and P107 H_α_, as well as cis-form P107 H_δ1_ and H_δ2_ (Figure 4B). This result indicated that TgCyp18 catalyzed the peptidyl-proline cis/trans isomerization specifically on proline residue P107 of the C-peptide.

We then extended this characterization to the entire TgPDCD5 protein. If TgCyp18 catalyzed the cis–trans isomerization of P107 in TgPDCD5, the intensities of the cross-peaks in the ^1^H-^15^N HSQC spectrum of TgPDCD5 would increase for residues in the cis-form and decrease for those in the trans form. Thus, we collected and compared the ^1^H-^15^N HSQC spectra of TgPDCD5 without and with TgCyp18 enzyme treatment (at a ratio of 1:0.01) (Figure 4C,D). The chemical shifts in the two spectra were similar (Figure 4C). However, the resonance intensities of the overall protein increased (Figure 4D), suggesting the enzyme stabilizes the amide exchange rate of the protein, which implies a reduction in the population difference between the trans and cis-forms of the protein. Importantly, the increased resonance intensities of residues surrounding cis-P107 indicated an expansion of the cis-form population. This phenomenon demonstrated the catalysis of isomerization on P107 of the entire TgPDCD5 protein, resulting in a more balanced homogeneity of the protein pool.

In the next step, we investigated whether the P107 residue could act as a proline switch to regulate the binding affinity of heparin sulfate. NMR chemical-shift perturbations (CSPs) of TgPDCD5 upon the addition of Enoxaparin were measured in the presence of TgCyp18 (at a ratio of 1:0.01) (Figure 4E,F). By calculating the difference in CSPs between the titrations with the absence (Figure 3G) and presence of TgCyp18 (Figure 4F) with the definition of ΔCSP (CSP_–Cyp18_ – CSP_+Cyp18_) (Figure 4G), we have the opportunity to assess the role of P107 isomerization in the binding of Enoxaparin with TgPDCD5. This exploration is feasible due to the consistent concentrations of TgPDCD5 and polysaccharides between the groups. Notably, the ΔCSP values of A62 were greatly reduced after the P107 isomer exchange of TgPDCD5 (Figure 4G), from 0.18 to 0.11 ppm. Moreover, the overall chemical shifts were decreased as shown in Figure 4G, especially those peaks corresponding to the C-terminal TgPDCD5 that had been highlighted with obvious CSP in Figure 3C. Interestingly, it appears that the increased population of the cis-form of TgPDCD5 resulted in reduced CSP effects on the residues in the core region as well as residues around the HSPG-binding motif.

To validate the role of P107 as a proline switch in regulating heparin binding, we introduced a point mutation, replacing proline with alanine, into the TgPDCD5 protein. The CSP profile of TgPDCD5_P107A_ upon the addition of Enoxaparin showed smaller changes compared to wild-type TgPDCD5 (Figure 4H–J). This result indicates that the absence of the proline switch in TgPDCD5_P107A_ leads to a consistently low binding affinity.

### Critical Residues of TgPDCD5 for Heparin Sulfate Binding

When we mapped the residues with significant chemical-shift differences onto the solution structure of TgPDCD5, we observed that these amino acids were located on the surface of the protein, extending through the core and C-terminus (Figure 3G). The Enoxaparin binding surface of the major trans-form of TgPDCD5 consisted of residues Q33, Q58, A62, D63, K64, E87, L94, A100, T106, M111, R112, R113, R114, S116, D117, D121, and F122. In contrast, the major cis-form Enoxaparin binding surface was formed by residues A62, E69, E87, L94, T106, M111, R112, R113, R114, S116, and D117. Among these residues, only M111, R112, and R113 were located in the HSPG-binding motif previously identified.^18^ This result suggests that TgPDCD5 utilizes not only the C-terminal HSPG-binding motif but also its core region to interact with heparin.

**Figure 5:**
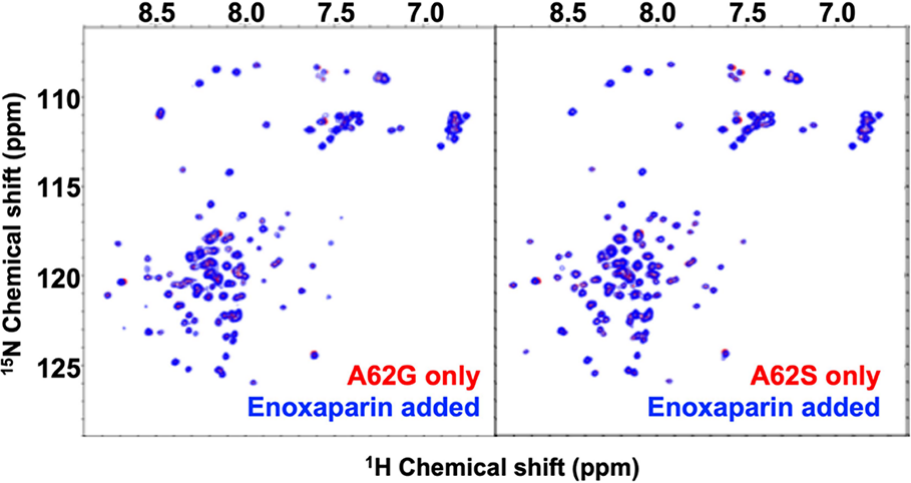
Critical residues involved in Enoxaparin binding. 2D ^1^H-^15^N HSQC spectrum of TgPDCD5 mutants A62G and A62S titrated with Enoxaparin, respectively. The red spectrum represents backbone amides of TgPDCD5 mutants before titration, while the blue spectrum represents backbone amides of TgPDCD5 mutants after titration with a ratio of 1:0.6 (protein:Enoxaparin).

Based on the results of our NMR perturbation assay, we introduced several mutations into the TgPDCD5 sequence (Figure S4A). Since the heparin-binding surface is broad and not site-specific, we designed an electrophoretic mobility shifting assay (EMSA) to roughly determine the Enoxaparin binding abilities of each mutation, without the need for protein purification (Figure S4B). The A62S and S116A, D117A, D121A, and F122A mutants showed significantly different Enoxaparin shifting patterns compared to the wild-type-expressed cell lysates of *E. coli* (* symbols in Figure S4B). Based on the results of the EMSA, along with our suspicions, eight mutated TgPDCD5 recombinant proteins were purified and subjected to NMR perturbation assays (Figure 5). The interaction between TgPDCD5 and Enoxaparin reached saturation at a protein-to-Enoxaparin ratio of 1:0.6, and the mutants were titrated with Enoxaparin under the same conditions. Clearly, the Enoxaparin binding ability of TgPDCD5 was completely blocked when A62 was mutated to glycine or serine. Previous studies on the heparin-binding hemagglutinin from *Mycobacterium tuberculosis*, which complexes with chemically synthesized heparan sulfate octasaccharide, suggested that alanine residues bind to the sugar rings through hydrophobic interactions.^37^ Based on this information, we speculated that the alanine residues in TgPDCD5 might interact with the ring structures in Enoxaparin via hydrophobic interactions. We also mutated another alanine, A100, located on the identified Enoxaparin binding surface. The results of Enoxaparin titration with the A100G or A100S mutants showed a reduction in Enoxaparin binding ability (Figure S6). It appeared that A62 was the most critical alanine involved in Enoxaparin binding. Residues M111 and R112, located in the previously predicted HSPG-binding motif,^18^ were also subjected to mutations. The chemical-shift differences were observed in Enoxaparin titrations to TgPDCD5_M111A_ and TgPDCD5_R112A_ but were smaller than those observed in the Enoxaparin titration to TgPDCD5, indicating that these two residues somehow play a role in the Enoxaparin interaction (Figure S6). The Enoxaparin binding surface of TgPDCD5 contains three consecutive arginine residues with positively charged side chains, which may interact with the polysaccharide via electrostatic effects. However, when we introduced quadruple mutations (M111A/R112A/R113A/R114A) to this position, the blockage of Enoxaparin binding did not occur as expected (Figure S6). Similarly, when we introduced quadruple mutations (S116A/D117A/D121A/F122A) near the TgPDCD5 C-terminus, consisting of consecutive aspartic acids, the Enoxaparin binding ability was only slightly decreased. Taken together, it appears that residue A62 is the most essential residue on the Enoxaparin binding surface. We further used another biophysical approach to confirm our suggestion (Figure S7), and no significant heat difference was observed during the ITC analysis when TgPDCD5_A62G_ or TgPDCD5_A62S_ was titrated with Enoxaparin. Based on the CSP analysis (Figures 4E–J and 3G) and mutagenesis results (Figure 5), we hypothesize that the major trans form of TgPDCD5 prefers to interact with heparin using its core region, while the involvement of the C-terminus is relatively minor.

**Figure 6:**
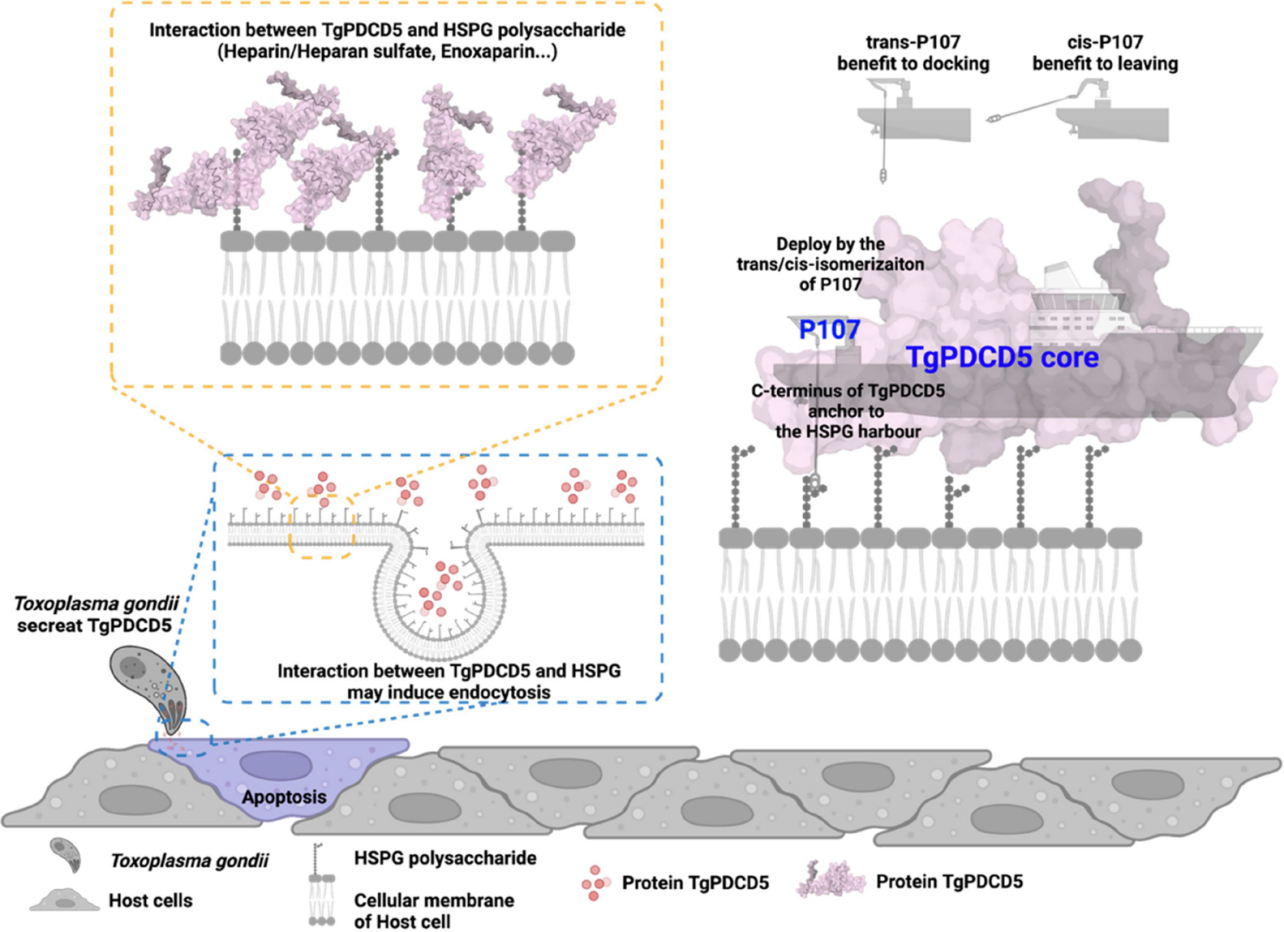
Mechanism of the interaction between TgPDCD5 and HS/heparin polysaccharide heparin. Structural mechanism of the interaction between the *T. gondii* tachyzoite secreted protein TgPDCD5 and HS/heparin polysaccharide. The *T. gondii* tachyzoite is represented by a gray crescent moon-shaped module, while the host cells are depicted as gray cloudy shapes. The purple host cell represents apoptosis induction. The host cell plasma membrane is shown as gray double layers with bean sprout-shaped phospholipid components. The gray bead streams on the plasma membrane represent HS/heparin polysaccharides. The pink pearls represent the TgPDCD5 protein, with its protein surface colored in pink.

Taken together, we propose an HSPG (heparin polysaccharide proteoglycan) binding mechanism for TgPDCD5 (Figure 6). The protein TgPDCD5 is likened to a boat, and HSPG or heparin acts as the harbor. The boat TgPDCD5 docks its core to the harbor, guided by the rope represented by the “C-terminal HSPG binding motif,” which can be switched by the anchor shackle P107. When the shackle P107 is in the trans-form, the interaction between TgPDCD5 and heparin is stronger, which may facilitate docking. Conversely, when the shackle P107 is in the cis-form, the binding to heparin is weaker, guiding the release of TgPDCD5. Our proposed mechanism is supported by a previous study on how proline, as a molecular switch, regulates protein binding in an intrinsically disordered protein, NCBD (the nuclear coactivator binding domain of CBP), through its cis/trans isomerization.^38^

The structural complexities, dynamic features, and challenging synthetic or purification methods of HS/heparin polysaccharides pose a challenge for researchers studying protein interactions with these molecules, whether from a biological, biophysical, or biochemical perspective. The concept of the HSPG-binding motif for HS recognition was established in 1989. Based on sequence analysis and molecular modeling, the rule of the HSPG-binding motif was defined as linear sequences of basic amino acids interspersed with other, often hydrophobic, amino acids.^39^ However, from the perspective of structural biologists, it is intriguing how a protein interacts with such a large polysaccharide macromolecule using only six amino acids. Our study provides robust evidence to describe a molecule with high flexibility due to its molten globular properties and the modulation of heparin sulfate interaction through proline isomerization. Nevertheless, further research is needed to establish the correlation between these molecular behaviors of TgPDCD5 and its biological functions.

## Conclusions

The concept of the MG state has traditionally been defined as a compact intermediate state in which the tertiary structure of the protein is disrupted while the secondary structure remains intact or even strengthened.^40^ In the early 1990s, the MG state gained significant interest due to its proposed similarity to intermediate states in protein folding, as well as its potential role as a translocation-competent state in the transport of precursor proteins across biological membranes.^41−44^ Here, our study provides comprehensive insights into the molecular properties and binding mechanism of TgPDCD5, a protein involved in heparin sulfate recognition and the modulation of host–pathogen interactions. Through a combination of biophysical techniques and functional assays, we demonstrate that TgPDCD5 exhibits a molten globular behavior and undergoes proline isomerization, which acts as a switch to regulate its binding affinity for heparin sulfate.

Our findings highlight the importance of both the core region and the C-terminal HSPG-binding motif of TgPDCD5 in heparin sulfate binding. We propose a model where TgPDCD5, like a boat, docks its core region to the harbor of heparin sulfate, utilizing the C-terminal motif as a rope that can be switched by proline residue P107. The conformational changes induced by proline isomerization modulate the binding affinity, allowing TgPDCD5 to interact with heparin sulfate in a dynamic and regulated manner. Furthermore, our study reveals the involvement of specific amino acid residues, particularly A62, in the Enoxaparin binding surface of TgPDCD5. Mutagenesis studies confirm the critical role of A62 in the binding affinity, suggesting that hydrophobic interactions between alanine residues and the sugar rings of Enoxaparin contribute to the binding specificity.

Overall, our research provides a deeper understanding of the intricate interactions between TgPDCD5 and heparin sulfate, shedding light on the molecular mechanisms underlying host–pathogen interactions. The elucidation of the binding mechanism and identification of key residues involved in the interaction opens up new avenues for the development of therapeutic strategies targeting these interactions. Further investigations into the biological functions and implications of TgPDCD5′s binding behavior will enhance our understanding of the host–pathogen relationship and may pave the way for the development of novel antiparasitic interventions.

In conclusion, our study uncovers the dynamic nature of TgPDCD5, revealing how it navigates the complex landscape of heparin sulfate recognition. These findings contribute to the broader field of host–pathogen interactions and provide a basis for further research and potential therapeutic applications.

## Materials and Methods

### Protein Expression and Purification

A DNA fragment encoding the TgPDCD5 sequence was synthesized with codon optimization and subsequently cloned into a pET-28a vector using NdeI and XhoI restriction enzyme sites for the *E. coli* expression system. A TEV protease recognition site (ENLYFQ↓S) was strategically positioned between the His-tag and TgPDCD5 to facilitate further tag removal. Recombinant TgPDCD5 with an N-terminal His-tag was expressed in *E. coli* BL21 (DE3) at an OD_600_ of 0.4–0.6 in LB (or M9 for NMR) medium at 16 °C, induced with 1 mM (isopropyl β-D-1-thiogalactopyranoside) IPTG for 16 h. The cells were harvested by centrifugation at 6,000 rpm for 10 min and resuspended in a lysis buffer containing 25 mM sodium phosphate, 1 M NaCl, pH 7.5, with 1 mM phenylmethanesulfonyl fluoride (PMSF). After sonication for cell lysis, the lysate was centrifuged at 13,000 rpm at 4 °C for 20 min to remove debris. The recombinant protein was purified using a Ni-NTA column pre-equilibrated with lysis buffer. His-tagged TgPDCD5 was eluted with 300 mM imidazole in a lysis buffer. Eluted fractions were dialyzed against a buffer containing 25 mM sodium phosphate and 100 mM NaCl, pH 7.5, and further purified using a heparin column with a NaCl concentration gradient ranging from 100 mM to 1M. Given the absence of aromatic amino acids such as tyrosine and tryptophan in the TgPDCD5 sequence, each fraction obtained from the heparin column purification underwent SDS-PAGE analysis, as there was no UV280 absorbance signal available. The purified protein fractions were collected and digested with 2 mg of homemade TEV protease (Addgene: 8827) to remove the His-tag during overnight dialysis at 4 °C with 3L of dialysis buffer (25 mM sodium phosphate and 100 mM NaCl). The homemade TEV protease containing a His-tag was removed through a second round of Ni-NTA column purification. TgPDCD5 protein was further purified using a size-exclusion column (Superdex75 XK 16/60, GE Healthcare). The purified protein was concentrated to a concentration of 0.1–0.5 mM in a buffer containing 25 mM sodium phosphate, 100 mM NaCl, 10 mM sodium azide, and 1 mM PMSF at pH 4.5 for NMR and in vitro assays. The pH value was adjusted to 6.5 for the host cell assays.

Recombinant TgCyp18 with an N-terminal GST-tag was expressed in *E. coli* BL21 (DE3) at OD_600_ of 0.6 in LB medium at 16 °C in the presence of 1 mM IPTG for 16 h. The cells were harvested by centrifugation at 6000 rpm for 10 min and resuspended in lysis buffer containing 25 mM sodium phosphate, 100 mM NaCl, pH 7.0, with 1 mM PMSF. After sonication for 20 min, the lysed cells were centrifuged at 13,000 rpm at 4 °C for 20 min to remove debris. The supernatant of the lysate was applied to a glutathione sepharose column pre-equilibrated with lysis buffer. The column was washed with 50 mL of lysis buffer. A solution containing 30 mg of thrombin powder mixed with 10 mL of lysis buffer was prepared. This thrombin solution was loaded onto the column, which was then sealed with a top cap and stopper. On-column thrombin cleavage was performed by incubating the column at 10 °C overnight. The following day, 5 mL fractions of tag-removed TgCyp18 were collected, while the column was washed with 30 mL of lysis buffer. The collected fractions were further purified using a size-exclusion column (Superdex75 XK 16/60, GE Healthcare). The remaining GST-tags were removed from the glutathione sepharose column by using 50 mM glutathione. The purified TgCyp18 protein was concentrated to a concentration of 1.5–3.0 mM in 25 mM sodium phosphate buffer with 100 mM NaCl (pH, 7 0) for further biochemical and NMR assays.

### CD Spectrometry Assays

CD spectra were measured using 10 μM protein samples in a 20 mM phosphate buffer at pH 2.5–6.5. The samples were placed into a 1 mm path length cuvette and recorded on a JASCO J-815 spectrometer. To determine the chemical denaturation, protein samples were preincubated with varying concentrations of urea before collecting CD spectra. The thermal transition of TgPDCD5 was monitored at 222 nm at a scan rate of 1 °C/min.

### SAXS Assays

Protein samples for SAXS were prepared in a buffer containing 25 mM phosphate at pH 6.5, 150 mM NaCl, and either no additives, 2 M, or 4 M urea. SAXS data were obtained using an online size-exclusion HPLC system at Beamline 23A, National Synchrotron Radiation Research Center, Taiwan. For each condition, 5 mg/mL TgPDCD5 protein was injected into a polymer-based HPLC column at a flow rate of 0.35 mL/min. Since TgPDCD5 lacks aromatic residues such as Y or W, the UV–vis absorption signals were weak. Therefore, HPLC size exclusion was monitored by an RI detector before X-ray exposure. The SAXS data were analyzed using the software Primus.^45^

### Fluorescence Spectroscopy

Fresh 10 mM 8-anilinonaphthalene-1-sulfonic acid (ANS), a fluorescence probe used to detect the exposure of the protein’s hydrophobic core,^46^ was dissolved in methanol and added to the protein solution to a final concentration of 40 μM. The final concentrations of the protein samples ranged from 162.5 to 650 μg/mL. A control mixture of ANS and the buffer without protein was also prepared. The excitation wavelength was set at 360 nm, and the spectra were recorded in the 400–600 nm region.

### High-Field Solution NMR

NMR experiments were performed on Bruker Avance 600, 800, or 850 MHz spectrometers at 310 K by using a 5 mm triple resonance cryoprobe and Z-gradient. The acquired data were processed using Topspin 3.6 software (Bruker, Germany) and further analyzed using CARA (http://cara.nmr.ch/doku.php/home, Keller). The ^1^H chemical shifts were referenced externally to 0 ppm using 2,2-dimethyl-2-silapentane-5-sulfonate as a standard. The ^15^N and ^13^C chemical shifts were indirectly referenced according to IUPAC recommendations.^47^ Protein backbone assignments were obtained through triple resonance experiments, including HNCACB, CBCA(CO)NH, HNCA, HNCOCA, HNCO, and HN(CA)CO. Side-chain assignments were accomplished by using HCCH-TOCSY experiments. For NMR structure calculation, the chemical-shift assignment, the NOE distance restraints, dihedral angles from the prediction of TALOS, information from hydrogen/deuterium exchange, and the RDC data collected with the Pf1-aligned phage were performed in software CYANA 3.0.

For the Enoxaparin titration, the appropriate amount of Enoxaparin was added to a 0.2 mM 15N-labeled protein solution, resulting in a final protein-to-Enoxaparin ratio of 1:0.6. The 1H-15N HSQC spectra were collected at 310 K during the titration. The chemical-shift differences between the backbone amide 1H and 15N resonances of TgPDCD5 in the presence and absence of Enoxaparin were calculated using the following equation^48^:
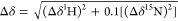


All NMR relaxation experiments were performed at 310 K on a Bruker Avance 600 MHz spectrometer. For *R*_1_ measurements, the relaxation delay times were set to 10, 600, 200, 70, 140, 400, 500, 1000, 300, and 800 ms. For *R*_2_ measurements, the relaxation delay times were set to 16.96, 84.8, 169.6, 0, 203.52, 50.88, 118.72, 152.64, 254.4, and 237.43 ms. NOE values were obtained by collecting data sets with and without 4 s of initial proton saturation. The acquired relaxation data were analyzed using Dynamicscenter 2.5.1 software.

The resonance assignment of the TgPDCD5 C-peptide (^104^KNTPKVTM^111^) was done by TOCSY at 298 K. The ^1^H-^1^H ROESY spectrum of the TgPDCD5 C-peptide (^104^KNTPKVTM^111^) with the absence (ratio 40:0) and presence (ratio 40:1) of enzyme Cyp18 were collected at 298 K on a Bruker Avance 800 MHz spectrometer.

### ITC Measurement

Quantitative estimations of heparin-binding affinities for some proteins using ITC have been reported.^49−51^ To investigate the interaction between TgPDCD5 and heparin sulfate (H3393, Sigma-Aldrich) or Enoxaparin (E0180000, Sigma-Aldrich), ITC was performed using a Nano ITC instrument (TA Instruments). Aliquots of 4 μL of 3 mM TgPDCD5 were injected into 0.12 mM heparin sulfate or Enoxaparin in 25 mM phosphate buffer at pH 4.5 with 100 mM NaCl while maintaining a temperature of 37 °C with 250 rpm stirring. Background heat from the protein to buffer titrations was subtracted. The thermal parameters (enthalpy Δ*H* and entropy Δ*S*), stoichiometry of the binding (*n*), and dissociation constant (*K*_d_) were derived by fitting the data to an independent binding model using Launch NanoAnalyze v2.3.6 software.

### MTT Assay

The MTT assay protocol from Abcam was followed for the experiments. U937 cells were cultured at a density of 2 × 105 cells/mL prior to the experiments. Various dilutions of recombinant proteins were added to the cell culture. After 24 h, 10 μL of MTT PBS solution (final concentration of 0.5 mg/mL) was added to each well. Following a 3 h incubation, the MTT solvent (4 mM HCl and 0.1% NP40 in isopropanol) was added. The absorbances were measured at 590 nm using an ELISA reader after 15 min of shaking.

### Flow Cytometry Measured Annexin V/PI Staining Assay

The Annexin V/PI staining assay was conducted using the Annexin V Apoptosis Kit-FITC (ApoScreen, SouthernBiotech) and analyzed by flow cytometry. U937 cells were cultured at a density of 2 × 105 cells/mL prior to the experiments. The appropriate amount of recombinant proteins was determined by using the MTT assay. Purified recombinant proteins at a concentration of 10 μg/mL were added to the cell culture. After 24 h, the cells were harvested by centrifugation at 5000 g for 10 min at 4 °C, and washed twice with cold PBS. The collected cells were resuspended in 200 μL of cold 1× Annexin binding buffer obtained from the commercial kit. Subsequently, 10 μL of conjugated Annexin V-FITC was added. The tubes were gently vortexed and incubated on ice for 15 min. To the reactions, 380 μL of Annexin binding buffer and 10 μL of propidium iodide (PI) were added. The reactions were then immediately analyzed by flow cytometry.

### FP Assay

To assess the prolyl-peptide-binding ability of TgCyp18, an FP assay was conducted. The FITC-labeled TgPDCD5 peptides were synthesized by a local company, Yao-Hong Biotechnology. Fifteen microliters of 5′ FITC-labeled TgPDCD5 peptides, prepared in 25 mM sodium phosphate, 100 mM NaCl (pH 7.0) with 0.04% Tween 20, were incubated with 15 μL of serially diluted TgCyp18 in 25 mM sodium phosphate, 100 mM NaCl (pH 7.0) at room temperature for 5 min. The final concentration of the peptides was 150 μM. FP measurements were performed on the SpectraMax iD5 multimode microplate reader (Molecular Devices, CA, USA) using a black 384-well microplate, with an excitation wavelength of 485 nm and an emission wavelength of 535 nm.

### EMSA for Polysaccharide

To roughly estimate the Enoxaparin binding of TgPDCD5 mutants, 1 μL of 1 mg/mL Enoxaparin was added to 8 μL of cell lysate obtained from *E. coli* expressing either the wild-type (WT) or the designed TgPDCD5 mutants. The cell lysates were freshly prepared by lysing 5 mL of *E. coli* BL21 cells, induced with 1 mM IPTG, at 37 °C overnight using 1 mL of B-PER bacteria protein extraction reagent. After incubating for 10 min at room temperature, 1 μL of DNA orange G loading dye was added. The entire 10 μL reaction mixture was loaded onto a 15% TBE EMSA gel. The samples were resolved for 4 h at 20 mA in TBE buffer. The EMSA gels were stained with Toluidine (0.1 g/100 mL methanol solution) for 10 min at room temperature and subsequently destained with water.

## Data Availability

Atomic coordinates of TgPDCD5 in trans and cis-forms of P107 was deposited with PDB accession codes: 8I25 and 8I26, respectively.

## Supporting Information Available

Backbones and side chains assignment of TgPDCD5, the dynamics parameters derived from reduced spectral density mapping, spectrometric results of TgPDCD5 core (45–100), host cellular viability measured with the treatment of TgPDCD5 proteins, designs for TgPDCD5 mutants, 2D ^1^H-^15^N HSQC spectrum of TgPDCD5 mutants, and result of ITC assay of TgPDCD5 A62G/S titrating with Enoxaparin (PDF)

## References

[ref1] Maenz M.; Schluter D.; Liesenfeld O.; Schares G.; Gross U.; Pleyer U. Ocular toxoplasmosis past, present and new aspects of an old disease. Prog. Retin Eye Res. 2014, 39, 77–106. 10.1016/j.preteyeres.2013.12.005. 24412517

[ref2] Martin S. Congenital toxoplasmosis. Neonatal Netw 2001, 20 (4), 23–30. 10.1891/0730-0832.20.4.23. 12143899

[ref3] Saadatnia G.; Golkar M. A review on human toxoplasmosis. Scand J. Infect Dis 2012, 44 (11), 805–814. 10.3109/00365548.2012.693197. 22831461

[ref4] Aleixo A. L.; Curi A. L.; Benchimol E. I.; Amendoeira M. R. Toxoplasmic Retinochoroiditis: Clinical Characteristics and Visual Outcome in a Prospective Study. PLoS Negl Trop Dis 2016, 10 (5), e0004685 10.1371/journal.pntd.0004685. 27136081 PMC4852945

[ref5] Morjaria S.; Epstein D. J.; Romero F. A.; Taur Y.; Seo S. K.; Papanicolaou G. A.; Hatzoglou V.; Rosenblum M.; Perales M. A.; Scordo M.; Kaltsas A. Toxoplasma Encephalitis in Atypical Hosts at an Academic Cancer Center.. Open Forum Infect Dis 2016, 3 (2), ofw070 10.1093/ofid/ofw070. 27096140 PMC4834739

[ref6] Soheilian M.; Ramezani A.; Azimzadeh A.; Sadoughi M. M.; Dehghan M. H.; Shahghadami R.; Yaseri M.; Peyman G. A. Randomized trial of intravitreal clindamycin and dexamethasone versus pyrimethamine, sulfadiazine, and prednisolone in treatment of ocular toxoplasmosis. Ophthalmology 2011, 118 (1), 134–141. 10.1016/j.ophtha.2010.04.020. 20708269

[ref7] Barragan A.; Sibley L. D. Migration of Toxoplasma gondii across biological barriers. Trends Microbiol. 2003, 11 (9), 426–430. 10.1016/s0966-842x(03)00205-1. 13678858

[ref8] Olafsson E. B.; Barragan A. The unicellular eukaryotic parasite Toxoplasma gondii hijacks the migration machinery of mononuclear phagocytes to promote its dissemination. Biol. Cell 2020, 112, 239 10.1111/boc.202000005. 32359185

[ref9] Sibley L. D. Intracellular parasite invasion strategies. Science 2004, 304 (5668), 248–253. 10.1126/science.1094717. 15073368

[ref10] Cabral C. M.; Tuladhar S.; Dietrich H. K.; Nguyen E.; MacDonald W. R.; Trivedi T.; Devineni A.; Koshy A. A. Neurons are the Primary Target Cell for the Brain-Tropic Intracellular Parasite Toxoplasma gondii. PLoS Pathog 2016, 12 (2), e1005447 10.1371/journal.ppat.1005447. 26895155 PMC4760770

[ref11] Koshy A. A.; Dietrich H. K.; Christian D. A.; Melehani J. H.; Shastri A. J.; Hunter C. A.; Boothroyd J. C. Toxoplasma co-opts host cells it does not invade. PLoS Pathog 2012, 8 (7), e1002825 10.1371/journal.ppat.1002825. 22910631 PMC3406079

[ref12] Nishikawa Y.; Kawase O.; Vielemeyer O.; Suzuki H.; Joiner K. A.; Xuan X.; Nagasawa H. Toxoplasma gondii infection induces apoptosis in noninfected macrophages: role of nitric oxide and other soluble factors. Parasite Immunol 2007, 29 (7), 375–385. 10.1111/j.1365-3024.2007.00956.x. 17576367

[ref13] Whitmarsh R. J.; Gray C. M.; Gregg B.; Christian D. A.; May M. J.; Murray P. J.; Hunter C. A. A critical role for SOCS3 in innate resistance to Toxoplasma gondii. Cell Host Microbe 2011, 10 (3), 224–236. 10.1016/j.chom.2011.07.009. 21925110 PMC3176442

[ref14] Brunet J.; Pfaff A. W.; Abidi A.; Unoki M.; Nakamura Y.; Guinard M.; Klein J. P.; Candolfi E.; Mousli M. Toxoplasma gondii exploits UHRF1 and induces host cell cycle arrest at G2 to enable its proliferation. Cell Microbiol 2008, 10 (4), 908–920. 10.1111/j.1462-5822.2007.01093.x. 18005238

[ref15] Chang S.; Shan X.; Li X.; Fan W.; Zhang S. Q.; Zhang J.; Jiang N.; Ma D.; Mao Z. Toxoplasma gondii Rhoptry Protein ROP16 Mediates Partially SH-SY5Y Cells Apoptosis and Cell Cycle Arrest by Directing Ser15/37 Phosphorylation of p53. Int. J. Biol. Sci. 2015, 11 (10), 1215–1225. 10.7150/ijbs.10516. 26327815 PMC4551757

[ref16] Bannai H.; Nishikawa Y.; Ibrahim H. M.; Yamada K.; Kawase O.; Watanabe J.; Sugimoto C.; Xuan X. Overproduction of the pro-apoptotic molecule, programmed cell death 5, in Toxoplasma gondii leads to increased apoptosis of host macrophages. J. Vet Med. Sci. 2009, 71 (9), 1183–1189. 10.1292/jvms.71.1183. 19801898

[ref17] Gavrilescu L. C.; Denkers E. Y. IFN-gamma overproduction and high level apoptosis are associated with high but not low virulence Toxoplasma gondii infection. J. Immunol 2001, 167 (2), 902–909. 10.4049/jimmunol.167.2.902. 11441097

[ref18] Bannai H.; Nishikawa Y.; Matsuo T.; Kawase O.; Watanabe J.; Sugimoto C.; Xuan X. Programmed Cell Death 5 from Toxoplasma gondii: a secreted molecule that exerts a pro-apoptotic effect on host cells. Mol. Biochem. Parasitol. 2008, 159 (2), 112–120. 10.1016/j.molbiopara.2008.02.012. 18406478

[ref19] Wiedemann C.; Bellstedt P.; Gorlach M. CAPITO--a web server-based analysis and plotting tool for circular dichroism data. Bioinformatics 2013, 29 (14), 1750–1757. 10.1093/bioinformatics/btt278. 23681122

[ref20] Lin M. H.; Yu T. A.; Chang C. F.; Nishikawa Y.; Hsu C. H. NMR resonance assignments of the programmed cell death protein 5 (PDCD5) from Toxoplasma gondii. Biomol NMR Assign 2020, 14 (2), 277–280. 10.1007/s12104-020-09961-8. 32578164

[ref21] Arai M.; Kuwajima K. Role of the molten globule state in protein folding. Adv. Protein Chem. 2000, 53, 209–282. 10.1016/S0065-3233(00)53005-8. 10751946

[ref22] Bychkova V. E.; Semisotnov G. V.; Balobanov V. A.; Finkelstein A. V. The Molten Globule Concept: 45 Years Later. Biochemistry 2018, 83 (Suppl 1), S33–S47. 10.1134/S0006297918140043. 29544429

[ref23] Judy E.; Kishore N. A look back at the molten globule state of proteins: thermodynamic aspects. Biophys Rev. 2019, 11 (3), 365–375. 10.1007/s12551-019-00527-0. 31055760 PMC6557940

[ref24] Khan M. K.; Rahaman H.; Ahmad F. Conformation and thermodynamic stability of pre-molten and molten globule states of mammalian cytochromes-c. Metallomics 2011, 3 (4), 327–338. 10.1039/c0mt00078g. 21431228

[ref25] Noga O.; Brunnee T.; Schaper C.; Kunkel G. Heparin, derived from the mast cells of human lungs is responsible for the generation of kinins in allergic reactions due to the activation of the contact system. Int. Arch Allergy Immunol 1999, 120 (4), 310–316. 10.1159/000024284. 10640915

[ref26] Oschatz C.; Maas C.; Lecher B.; Jansen T.; Bjorkqvist J.; Tradler T.; Sedlmeier R.; Burfeind P.; Cichon S.; Hammerschmidt S.; et al. Mast cells increase vascular permeability by heparin-initiated bradykinin formation in vivo. Immunity 2011, 34 (2), 258–268. 10.1016/j.immuni.2011.02.008. 21349432

[ref27] Meneghetti M. C.; Hughes A. J.; Rudd T. R.; Nader H. B.; Powell A. K.; Yates E. A.; Lima M. A. Heparan sulfate and heparin interactions with proteins. J. R Soc. Interface 2015, 12 (110), 20150589 10.1098/rsif.2015.0589. 26289657 PMC4614469

[ref28] Rabenstein D. L. Heparin and heparan sulfate: structure and function. Nat. Prod Rep 2002, 19 (3), 312–331. 10.1039/b100916h. 12137280

[ref29] Ahl I. M.; Jonsson B. H.; Tibell L. A. Thermodynamic characterization of the interaction between the C-terminal domain of extracellular superoxide dismutase and heparin by isothermal titration calorimetry. Biochemistry 2009, 48 (41), 9932–9940. 10.1021/bi900981k. 19754153

[ref30] Feng X.; Cheng Y.; Yang K.; Zhang J.; Wu Q.; Xu T. Host-guest chemistry of dendrimer-drug complexes. 5. Insights into the design of formulations for noninvasive delivery of heparin revealed by isothermal titration calorimetry and NMR studies. J. Phys. Chem. B 2010, 114 (34), 11017–11026. 10.1021/jp105958j. 20695473

[ref31] Zeng Z.; Patel J.; Lee S. H.; McCallum M.; Tyagi A.; Yan M.; Shea K. J. Synthetic polymer nanoparticle-polysaccharide interactions: a systematic study. J. Am. Chem. Soc. 2012, 134 (5), 2681–2690. 10.1021/ja209959t. 22229911 PMC3275679

[ref32] Eleraky N. E.; Swarnakar N. K.; Mohamed D. F.; Attia M. A.; Pauletti G. M. Permeation-Enhancing Nanoparticle Formulation to Enable Oral Absorption of Enoxaparin. AAPS PharmSciTech 2020, 21 (3), 88 10.1208/s12249-020-1618-2. 32016650

[ref33] Melis C.; Bussi G.; Lummis S. C.; Molteni C. Trans-cis switching mechanisms in proline analogues and their relevance for the gating of the 5-HT3 receptor. J. Phys. Chem. B 2009, 113 (35), 12148–12153. 10.1021/jp9046962. 19663504 PMC2733763

[ref34] Ibrahim H. M.; Xuan X.; Nishikawa Y. Toxoplasma gondii cyclophilin 18 regulates the proliferation and migration of murine macrophages and spleen cells. Clin Vaccine Immunol 2010, 17 (9), 1322–1329. 10.1128/CVI.00128-10. 20660134 PMC2944453

[ref35] Aliberti J.; Valenzuela J. G.; Carruthers V. B.; Hieny S.; Andersen J.; Charest H.; Reise Sousa C.; Fairlamb A.; Ribeiro J. M.; Sher A. Molecular mimicry of a CCR5 binding-domain in the microbial activation of dendritic cells. Nat. Immunol. 2003, 4 (5), 485–490. 10.1038/ni915. 12665855

[ref36] Dunyak B. M.; Gestwicki J. E. Peptidyl-Proline Isomerases (PPIases): Targets for Natural Products and Natural Product-Inspired Compounds. J. Med. Chem. 2016, 59 (21), 9622–9644. 10.1021/acs.jmedchem.6b00411. 27409354 PMC5501181

[ref37] Huang T. Y.; Irene D.; Zulueta M. M.; Tai T. J.; Lain S. H.; Cheng C. P.; Tsai P. X.; Lin S. Y.; Chen Z. G.; Ku C. C.; et al. Structure of the Complex between a Heparan Sulfate Octasaccharide and Mycobacterial Heparin-Binding Hemagglutinin. Angew. Chem., Int. Ed. Engl. 2017, 56 (15), 4192–4196. 10.1002/anie.201612518. 28294485

[ref38] Zosel F.; Mercadante D.; Nettels D.; Schuler B. A proline switch explains kinetic heterogeneity in a coupled folding and binding reaction. Nat. Commun. 2018, 9 (1), 3332 10.1038/s41467-018-05725-0. 30127362 PMC6102232

[ref39] Cardin A. D.; Weintraub H. J. Molecular modeling of protein-glycosaminoglycan interactions. Arteriosclerosis 1989, 9 (1), 21–32. 10.1161/01.ATV.9.1.21. 2463827

[ref40] Kuwajima K. The molten globule state as a clue for understanding the folding and cooperativity of globular-protein structure. Proteins 1989, 6 (2), 87–103. 10.1002/prot.340060202. 2695928

[ref41] Bychkova V. E.; Dujsekina A. E.; Klenin S. I.; Tiktopulo E. I.; Uversky V. N.; Ptitsyn O. B. Molten globule-like state of cytochrome c under conditions simulating those near the membrane surface. Biochemistry 1996, 35 (19), 6058–6063. 10.1021/bi9522460. 8634247

[ref42] Bychkova V. E.; Pain R. H.; Ptitsyn O. B. The ‘molten globule’ state is involved in the translocation of proteins across membranes?. FEBS Lett. 1988, 238 (2), 231–234. 10.1016/0014-5793(88)80485-X. 3049159

[ref43] Martin J.; Langer T.; Boteva R.; Schramel A.; Horwich A. L.; Hartl F. U. Chaperonin-mediated protein folding at the surface of groEL through a ‘molten globule’-like intermediate. Nature 1991, 352 (6330), 36–42. 10.1038/352036a0. 1676490

[ref44] van der Goot F. G.; González-Mañas J. M.; Lakey J. H.; Pattus F. A ‘molten-globule’ membrane-insertion intermediate of the pore-forming domain of colicin A. Nature 1991, 354 (6352), 408–410. 10.1038/354408a0. 1956406

[ref45] Hopkins J. B.; Gillilan R. E.; Skou S. BioXTAS RAW: improvements to a free open-source program for small-angle X-ray scattering data reduction and analysis. J. Appl. Crystallogr. 2017, 50 (Pt 5), 1545–1553. 10.1107/S1600576717011438. 29021737 PMC5627684

[ref46] Gasymov O. K.; Glasgow B. J. ANS fluorescence: potential to augment the identification of the external binding sites of proteins. Biochim. Biophys. Acta 2007, 1774 (3), 403–411. 10.1016/j.bbapap.2007.01.002. 17321809 PMC2039916

[ref47] Markley J. L.; Bax A.; Arata Y.; Hilbers C. W.; Kaptein R.; Sykes B. D.; Wright P. E.; Wuthrich K. Recommendations for the presentation of NMR structures of proteins and nucleic acids. IUPAC-IUBMB-IUPAB Inter-Union Task Group on the Standardization of Data Bases of Protein and Nucleic Acid Structures Determined by NMR Spectroscopy. J. Biomol NMR 1998, 12 (1), 1–23. 10.1023/A:1008290618449. 9729785

[ref48] Farmer B. T. 2nd; Constantine K. L.; Goldfarb V.; Friedrichs M. S.; Wittekind M.; Yanchunas J. Jr.; Robertson J. G.; Mueller L. Localizing the NADP+ binding site on the MurB enzyme by NMR. Nat. Struct. Biol. 1996, 3 (12), 995–997. 10.1038/nsb1296-995. 8946851

[ref49] Powell A. K.; Yates E. A.; Fernig D. G.; Turnbull J. E. Interactions of heparin/heparan sulfate with proteins: appraisal of structural factors and experimental approaches. Glycobiology 2004, 14 (4), 17R–30R. 10.1093/glycob/cwh051. 14718374

[ref50] Dong J.; Peters-Libeu C. A.; Weisgraber K. H.; Segelke B. W.; Rupp B.; Capila I.; Hernaiz M. J.; LeBrun L. A.; Linhardt R. J. Interaction of the N-terminal domain of apolipoprotein E4 with heparin. Biochemistry 2001, 40 (9), 2826–2834. 10.1021/bi002417n. 11258893

[ref51] Gupta S.; Tiwari N.; Verma J.; Waseem M.; Subbarao N.; Munde M. Estimation of a stronger heparin binding locus in fibronectin domain III14using thermodynamics and molecular dynamics. RSC Adv. 2020, 10 (34), 20288–20301. 10.1039/D0RA01773F. 35520402 PMC9054198

