# Supplementary material for: Proline Isomerization and Molten Globular Property of TgPDCD5 Secreted from Toxoplasma gondii Confers Its Regulation of Heparin Sulfate Binding
Source: JACS Au. 2024 Mar 20;4(5):1763–74. doi: 10.1021/jacsau.3c00577 (PMC11134355; doi:10.1021/jacsau.3c00577)
Supplement: Supplementary file 1 — au3c00577_si_001.pdf [file au3c00577_si_001.pdf]

# Supporting Information

**Proline isomerization and molten globular property of TgPDCD5 secreted from *Toxoplasma gondii* confers its regulation of heparin sulfate binding**


Gloria Meng-Hsuan Lin[1,2,#], Tsun-Ai Yu[3], Chi-Fon Chang[3], Chun-Hua Hsu[1,2,4,*]

[1]*Department of Agricultural Chemistry, National Taiwan University, Taipei 10617, Taiwan,*

[2]*Genome and Systems Biology Degree Program, National Taiwan University and Academia Sinica, Taipei 10617, Taiwan,*

[3]*Genomic Research Center, Academia Sinica, Taipei 115201, Taiwan*,

[4]*Institute of Biochemical Sciences, National Taiwan University, Taipei 115201, Taiwan,*

[#]Present address: Department of Microbiology and Molecular Medicine, University of Geneva, Switzerland

*Correspondence author: Chun-Hua Hsu, andyhsu@ntu.edu.tw

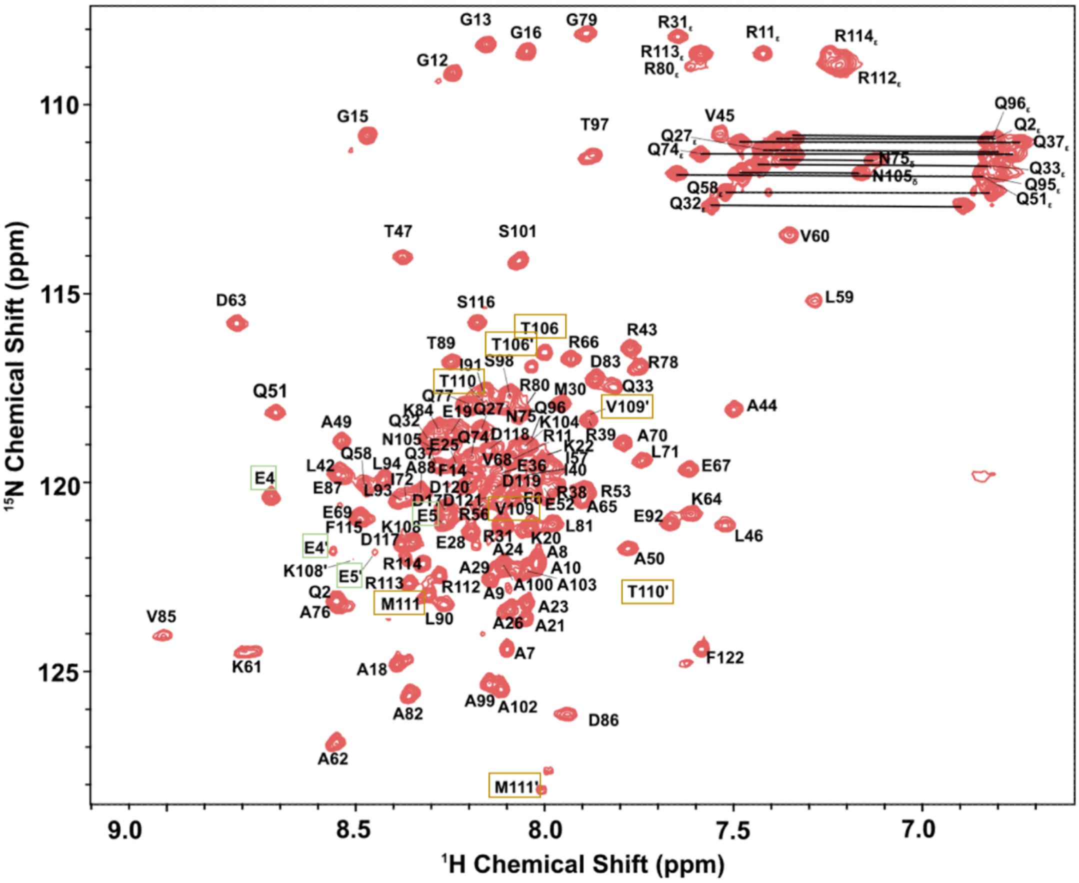


**Figure S1. Backbones and side chains assignment of TgPDCD5.**

The $^{1}$H-$^{15}$N HSQC spectrum of TgPDCD5 is shown with assigned $^{1}$H and $^{15}$N cross peaks presenting the backbone amides. The horizontal lines connect the side chain protons from amino acids N and Q. Side chain protons from R residues are also presented. Two sets of cis/trans peaks for P3 and P107 are highlighted in green and orange, respectively.

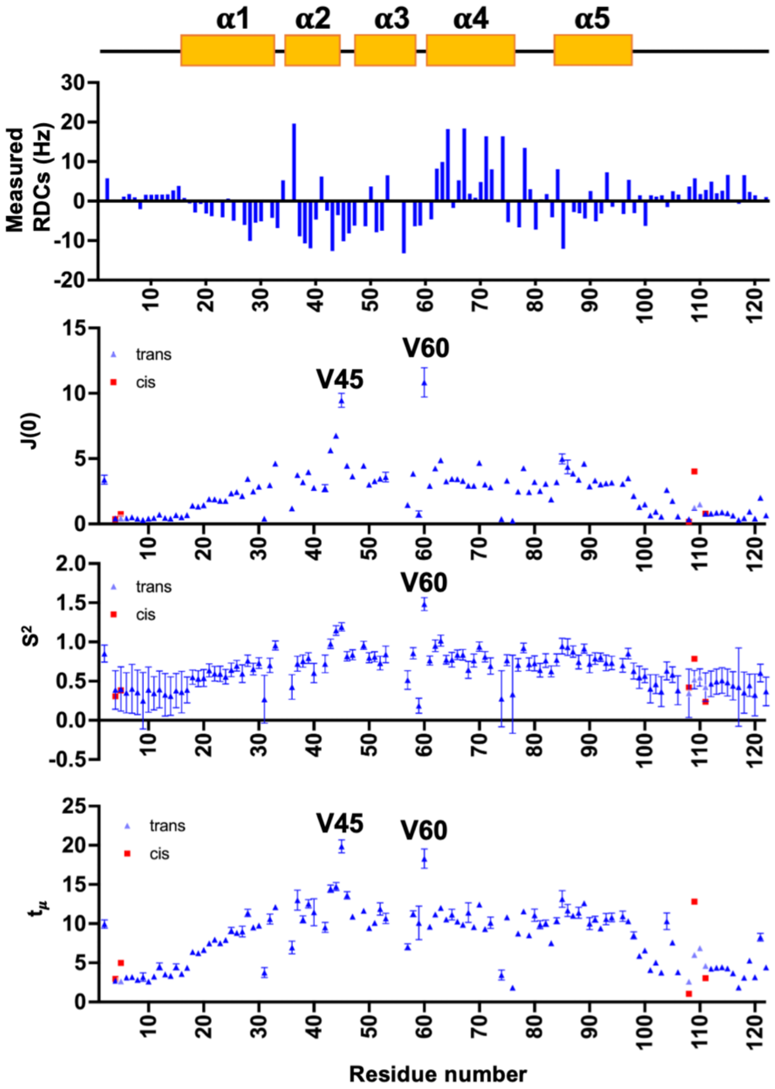


**Figure S2. The dynamics parameters derived from reduced spectral density mapping.**

Intramolecular dynamics information from amide RDCs measurements and reduced spectral density function mapping (J(0), $S^2$, and $t_\mu$) of TgPDCD5 are presented. Residues in the trans form are indicated by blue bars or triangles, and residues in the cis form are indicated by red squares.

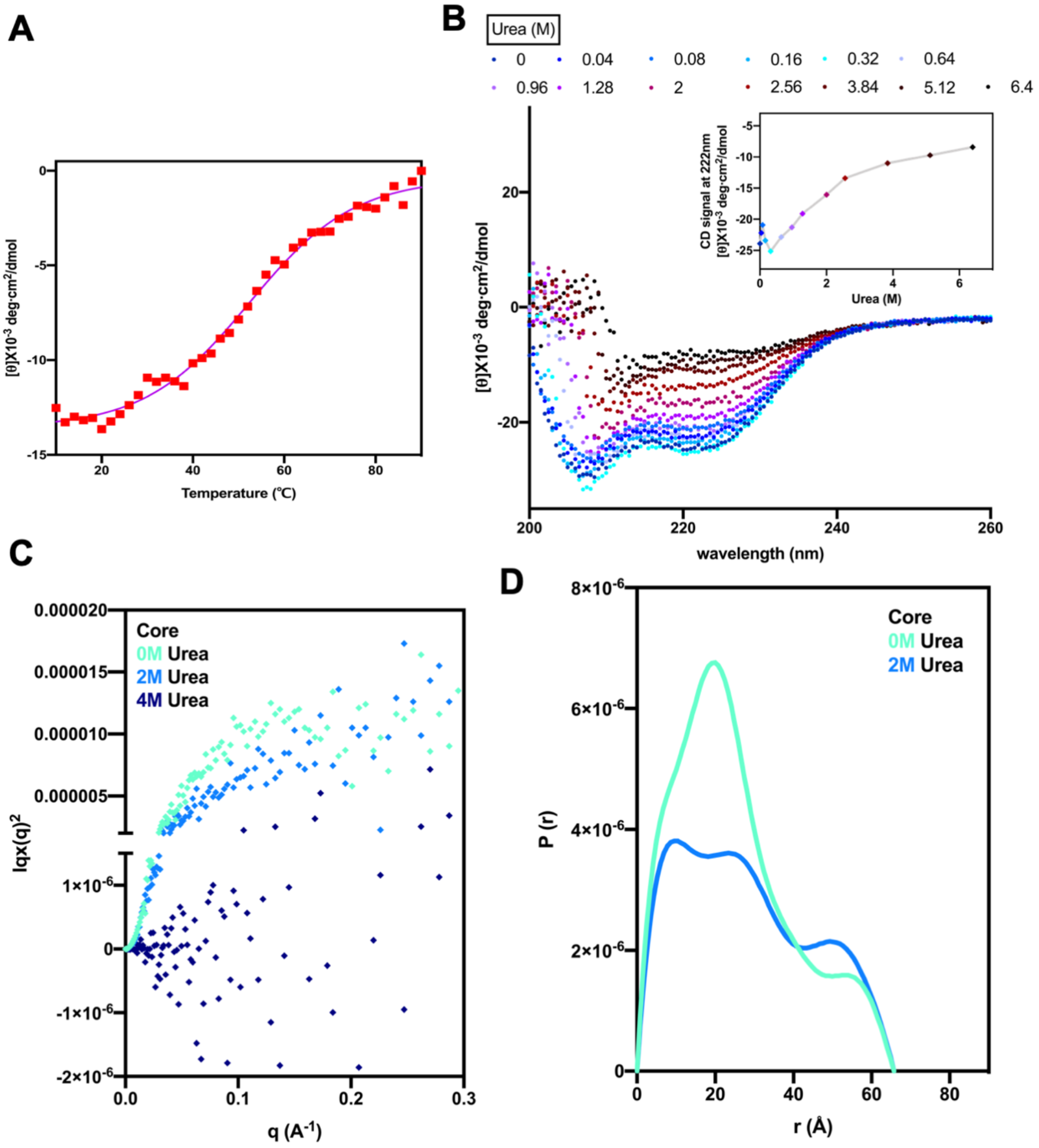


**Figure S3. Spectrometric results of TgPDCD5 core (45-100).**

(A) Thermal denaturation of protein TgPDCD5 (45-100) is shown, and the thermal-induced CD signal differences are fitted using a sigmoid model with software Prism and presented with a purple line. (B) Chemical denaturation of protein TgPDCD5 (45-100) is shown. (C) The Kartky-Porod plot of TgPDCD5 (45-100) chemical unfolding by urea is presented based on SAXS data. (D) The P(r) versus r profiles from the SAXS data are shown.

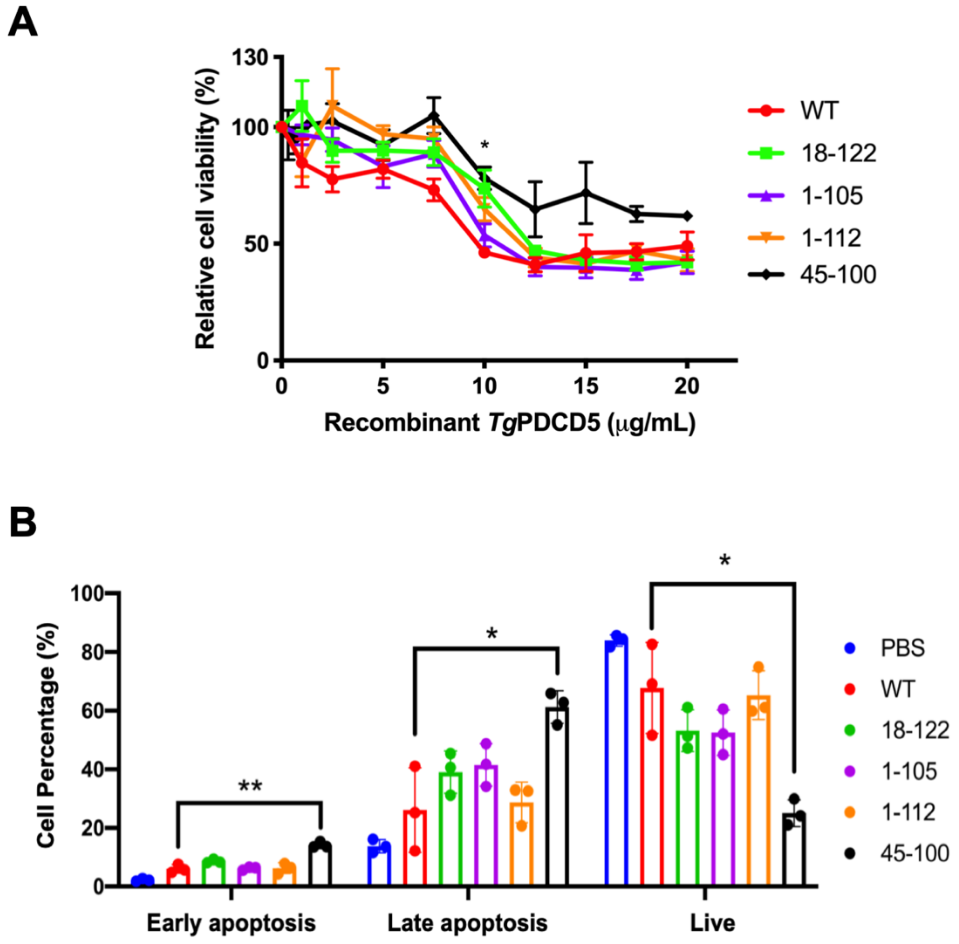


**Figure S4. Host cellular viability measured with the treatment of TgPDCD5 proteins.**

(A) U937 cell viability measured by MTT assay. Various concentrations of WT or truncations of TgPDCD5 (μg/mL) are added to U937 cell culture. After 24 hours, U937 cell viability is measured by MTT assay. Data represent the mean of three independent experiments. The concentrations of recombinant protein that significantly separate each group are labeled with an asterisk symbol (*). (B) Apoptosis induction by each fragment of TgPDCD5 measured by flow cytometry. Bars in red, green, purple, orange, black, and blue represent the apoptosis or live state of U937 cells after adding 10 μg/mL of WT TgPDCD5, TgPDCD5 fragment 18-122, 1-105, 1-112, 45-100, and PBS, respectively.

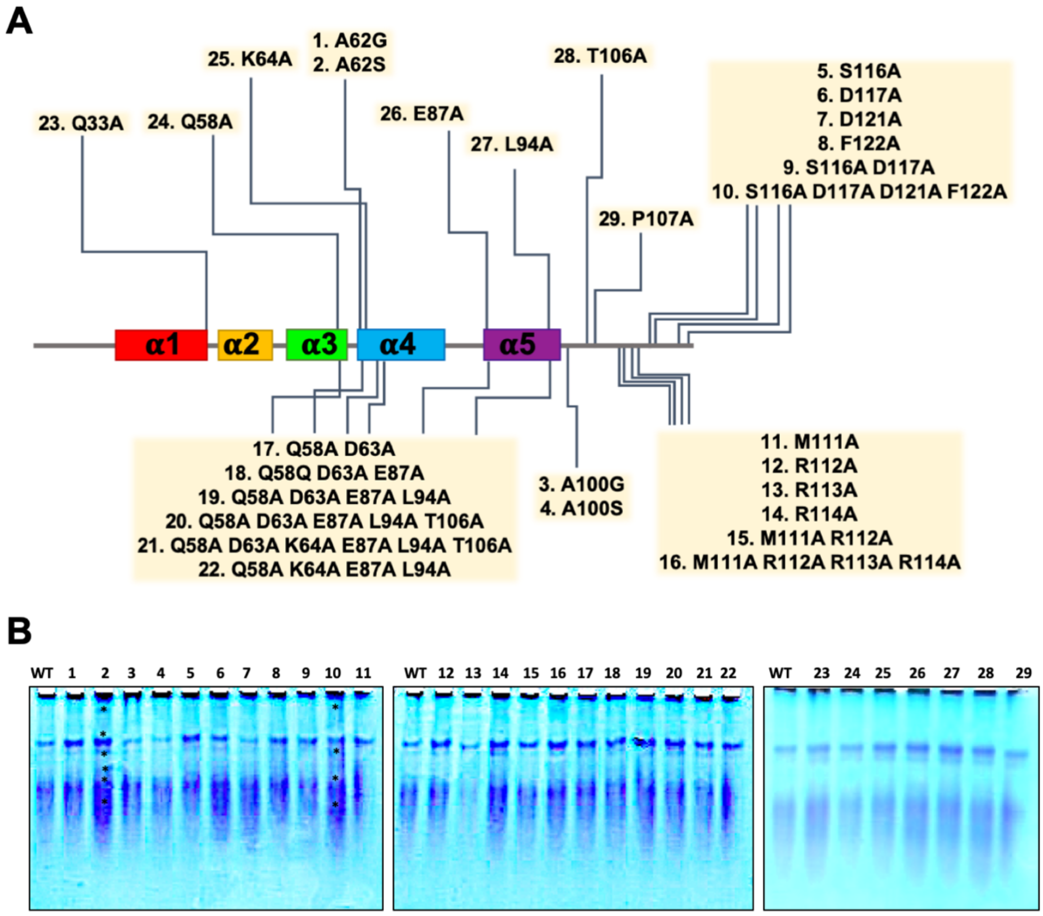


**Figure S5. Designs for TgPDCD5 mutants.**

(A) TgPDCD5 mutated positions, as determined by Enoxaparin titration CSPs, are illustrated. (B) Electrophoretic mobility shifting assay (EMSA) to assess the Enoxaparin binding abilities of each mutation. To enhance visibility of faint bands, the contrast and brightness of EMSA images have been adjusted.

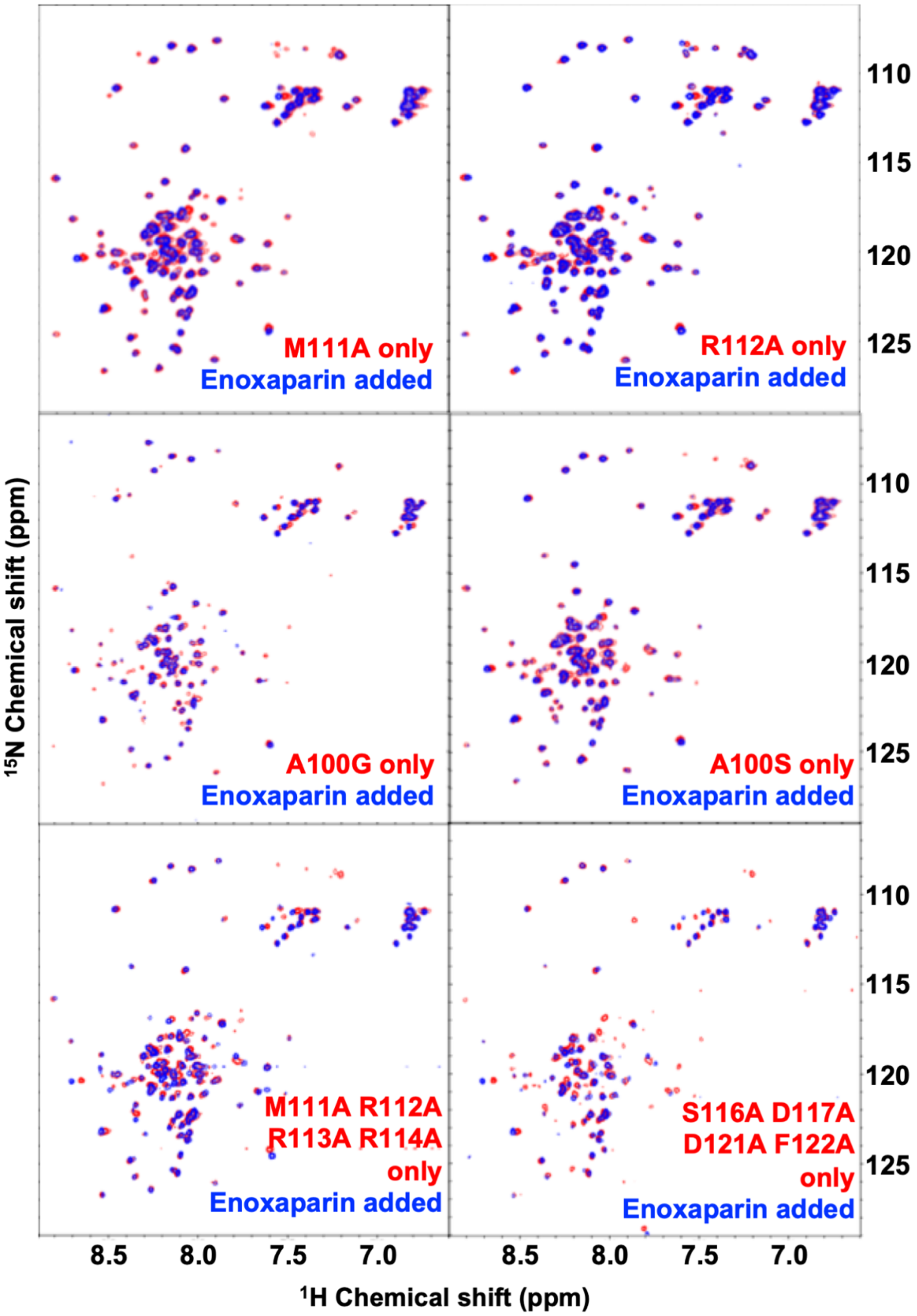


**Figure S6. 2D $^{1}H$-$^{15}N$ HSQC spectrum of TgPDCD5 mutants.**

Mutants include M111A, R112A, A100G, A100S, quadruple mutations in the HSPG-binding motif (M111A/R112A/R113A/R114A), and quadruple mutations at the C-terminus (S116A/D117A/D121A/F122A). Each mutant was titrated with Enoxaparin individually. Red cross-peaks represent the backbone amides of TgPDCD5 mutants before titration, while blue cross-peaks represent backbone amides of TgPDCD5 mutants after titration with a ratio of 1:0.6 (protein:Enoxaparin).

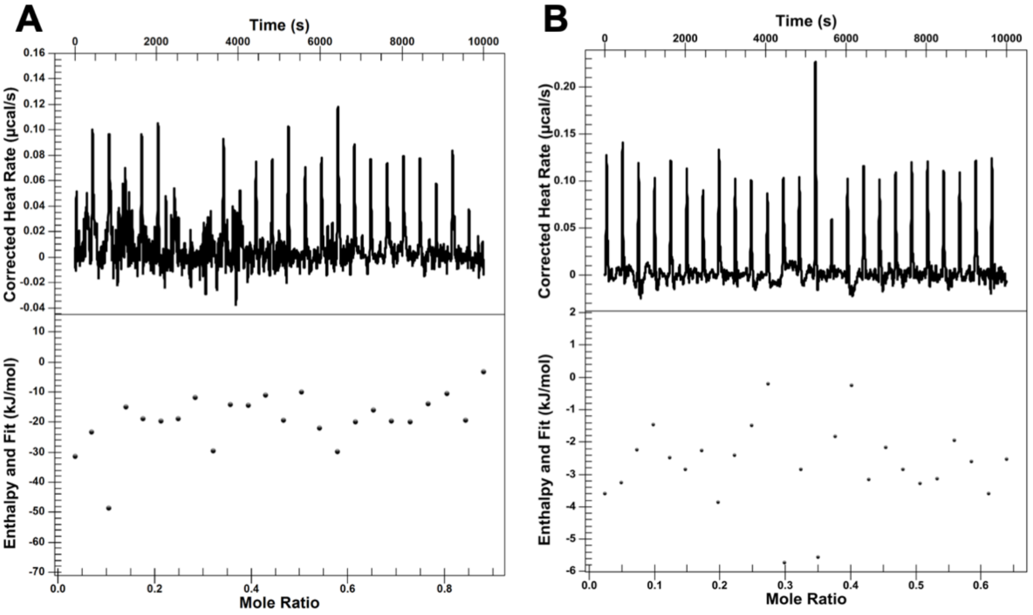


**Figure S7. Result of ITC assay of TgPDCD5 A62G/S titrating with Enoxaparin.** (A) Isothermal titration calorimetry analysis of TgPDCD5 A62G titrating into Enoxaparin. Upper panel: raw data in μcal/s versus time showing heat release during titration. Lower panel: integration of raw data yielding the heat per mole versus molar ratio. (B) Isothermal titration calorimetry analysis of TgPDCD5 A62S titrating into Enoxaparin. Upper panel: raw data in μcal/s versus time showing heat release during titration. Lower panel: integration of raw data yielding the heat per mole versus molar ratio.
